# Understanding lifestyle self-management regimens that improve the life quality of people living with multiple sclerosis: a systematic review and meta-analysis

**DOI:** 10.1186/s12955-022-02046-1

**Published:** 2022-11-25

**Authors:** Olivia C. Wills, Yasmine C. Probst

**Affiliations:** School of Medical, Indigenous and Health Sciences, University of Wollongong, Northfields Avenue, Wollongong, NSW 2522 Australia

**Keywords:** Multiple sclerosis, Self-management, Quality of life, Self-care, Lifestyle

## Abstract

**Background:**

Lifestyle self-management as an intervention for people living with multiple sclerosis (plwMS) is an emerging area of research. Previous reviews have highlighted a need to systematically identify effective self-management regimens that influence the health and well-being of plwMS using a common metric of success.

**Objectives:**

To examine the effectiveness of lifestyle self-management strategies and interventions aimed at improving the quality of life (QOL), and/or disability of plwMS. The review also aimed to narratively explore common elements of self-management interventions that were effective at improving the outcomes of interest.

**Methods:**

A systematic search was performed using five scientific databases. The review process followed the Cochrane Handbook for Systematic Reviews of Interventions  and was registered with PROSPERO (Ref: CRD42021235982).

**Results:**

A total of 57 studies including 5830 individuals diagnosed with MS, met the inclusion criteria. Self-management interventions included physical activity, fatigue, dietary, stress/coping, emotional, symptom and medical management, and lifestyle and wellbeing programs. Self-reported QOL improved in 35 of 47 studies. Dietary intervention had no statistically significant overall effect on reducing MS disability, (*P* = 0.18). Heterogeneity limited the ability to pool the effects from a large number of eligible studies of the same design.

**Conclusion:**

Multicomponent self-management interventions, multimodal delivery methods, and cognitive behavioural theory principles were common elements of self-management interventions that improved the QOL of plwMS. However, these results should be interpreted with caution and care should be taken in its clinical application. This review has the potential to inform future management practices for plwMS and has revealed a significant gap in the literature, warranting high-quality, large-scale experimental, and observational studies that address lifestyle management.

**Supplementary Information:**

The online version contains supplementary material available at 10.1186/s12955-022-02046-1.

## Introduction

### Rationale

Living with multiple sclerosis (MS) is associated with unpredictable and debilitating neurological symptoms [1]. These symptoms include physical challenges (i.e. fatigue, pain, weakness, spasticity), cognitive deficits (i.e. memory loss, slow processing, poor concentration), and/or emotional symptoms (i.e. anxiety, mood swings, irritability, depression) that fluctuate throughout the disease course, regardless of the MS phenotype [1, 2]. While medical management, including the provision of disease-modifying therapies (DMT’s), is important to maintain good health when living with MS, the impact of these symptoms is not restricted to routine healthcare visits. For most people living with MS (plwMS), these symptoms are experienced daily and create a multitude of challenges that can significantly impact quality of life (QOL) [2, 3]. Therefore, it is the day-to-day management of the disease course and symptoms that can have a profound impact on the current and future health and well-being of plwMS.

Self-management is a relatively new phenomenon within healthcare but has received increased attention as an effective management strategy for chronic health conditions; it is the ideology that acknowledges neurological conditions, such as MS, as a continuous experience [4]. Self-management focuses on equipping the individual with the skills and ability to manage their symptoms, monitor medication regimens and physical disability, engage in physical activity, maintain nutritional status, and adjust to the psychological demands of their condition [5, 6]. There is considerable evidence that self-management interventions are effective in improving the health and functional status, knowledge, adherence to treatment, and the physical, psychological, and social domains of QOL among people with chronic conditions [7–9]. The emergence of lifestyle self-management as an intervention for plwMS is a growing area of focus, and while currently limited, the effectiveness of self-management interventions holds promise [10].

A systematic review conducted by Kidd et al. examined the impact of self-management interventions on the psychological well-being of plwMS [11]. Despite the inclusion criteria, the limited number of published studies specific to MS care at the time (*n* = 10) and the heterogeneity between the included studies made it difficult to compare the effectiveness of different treatment strategies on self-reported outcome measures. Similarly, a second review exploring self-management support in people with neurological conditions generally, also concluded that the limited number of studies (*n* = 39) and the diversity in reported self-management outcome measures challenged the ability to comment on the overall effectiveness of the strategies [12]. More recently, Yamaguchi et al. examined the potential of self-management for people living with HTLV-1 associated myelopathy by reviewing the literature within an MS context. Again, the included studies reported inconsistent outcome measures (i.e. QOL, self-efficacy, cognitive changes) and only a limited range of medical, role (i.e. social life and independence) and emotional management strategies [13].

Previous reviews [11–13], therefore, highlight a need to systematically identify effective elements of self-management regimens that influence the health and well-being of plwMS using a common metric of success. QOL was chosen as an accepted outcome measure in clinical studies and has been identified as an important measure in MS research as it incorporates the personal and social context of a person’s life [14]. Moreover, Mitchell et al. recommended that studies of interventions in MS populations use assessments of QOL rather than impairment or disability measures alone [15].

### Aims and objectives

Therefore, the present review aimed to examine the effectiveness of lifestyle self-management strategies and interventions aimed at improving QOL, as the primary outcome, and/or disability among plwMS [14, 15]. The review also aimed to narratively explore common elements of self-management interventions that were effective at improving the outcomes of interest.

## Methods

### Registration and protocol

The present review was guided by the Cochrane Handbook for Systematic Reviews of Interventions [16] and reported according to the Preferred Reporting Items for Systematic Reviews and Meta Analysis (PRISMA) 2020 statement [17] and 27-item PRISMA checklist [17]. The review protocol was registered with the International Prospective Register of Systematic Reviews (PROSPERO) [18] (Ref: CRD42021235982) in February 2021, to reduce the potential for bias through a transparent review process [19].

### Eligibility criteria

Studies were included in the review if they met the following criteria:

*Types of studies and publications* Studies published in peer-reviewed journals, including both experimental and observational studies. Non-English studies were excluded. Studies including case studies, review articles, feasibility and pilot studies, protocols, grey literature, and publications including conference abstracts, editorials, and monographs were also excluded. No date restrictions were applied to the final search.

*Types of participants* Adults aged $\ge 18$ years with a clinical diagnosis of MS using the McDonald criteria (2001, 2005, 2010, or 2017) [20] regardless of their time since diagnosis. Studies that included people diagnosed with a clinically isolated syndrome phenotype, pregnant or breastfeeding females, or paediatric MS were excluded.

*Types of interventions* A study that described a self-management intervention followed by a participant was included. A self-management intervention was defined as a strategy that provided the individual with the opportunity to learn self-management skills by focusing on the tasks and/or healthy behaviour(s) in a person’s life, supporting them to accomplish a task, and assist in the management of their condition. The MS Research Australia Modifiable Lifestyle Factors guidance documents were used to classify self-management interventions [21]. Therefore, elements of self-management included any combination of symptom management strategies, diet and nutrition, physical activity and exercise, stress management, fatigue management, meditation/relaxation therapy, psychological management, rehabilitation strategies, supplementation use, and sun exposure [21].

*Types of comparators* Any comparator was considered for inclusion, and studies with no control groups were also included. The control arms of the intervention studies included participants who did not receive treatment or received usual care.

*Types of outcomes* Studies that described QOL as a primary outcome, or disability as a primary or secondary outcome, using a validated measure were included. This included, but was not limited to, the 36-item Short Form Survey (SF-36), multiple sclerosis impact scale (MSIS-29), multiple sclerosis quality of life-54 (MSQOL-54), quality of life-3 (QOL-3), short form-12 (SF-12), short form-8 (SF-8), Euro-quality of life, Hamburg quality of life questionnaire MS (HAQUAMS), and the World Health Organization QOL instrument (WHOQOL) [22].

Measures of disability included the expanded disability status scale (EDSS) and patient-determined disease steps (PDDS). The EDSS is a clinically administered assessment scale, and plwMS are assigned a score that corresponds to their level of ambulatory ability (ranging from 0: normal neurological exam/no disability to 10: death due to MS) in 0.5 increments [23, 24]. The PDDS is a self-reported tool to identify the level of physical disability in plwMS, which strongly correlates with the EDSS [25]. The tool is scored on a scale from 0 (normal; functionally normal with no limitations on lifestyle) to 8 (bedridden). Additional measures of disability included lesion burden (number of new lesions, size of lesions) as measured by MRI, number of relapses in a certain time period (relapse rate), and Guy’s neurological disability scale [26].

### Search strategy

The research question in the PICO format was: What is the effect of lifestyle self-management strategies and/or interventions on QOL and/or disability in plwMS? A systematic search was conducted using five scientific databases: Cochrane Library, CINAHL via EBSCOhost platform, MEDLINE via Ovid platform, PubMed via ProQuest platform, and Scopus via Elsevier. Although PubMed is a subset of MEDLINE, both MEDLINE and PubMed databases were searched to ensure that the most recent studies were identified [27]. A preliminary search was performed in February 2021 to determine the feasibility of the search by identifying sentinel articles. The final search was conducted on 8 April 2021 (OW).

The search strategy included alternative phrases, spelling, and truncations, using both controlled vocabulary and free-text terms. For a detailed search strategy, please refer to Additional file 1: data 1. To increase the sensitivity, manual hand searching of the reference lists of extracted systematic reviews and meta-analyses was performed to obtain additional relevant studies.

### Selection process

A three-phase screening process was implemented to ensure that the included studies met eligibility criteria. All studies were exported to Covidence (Covidence Systematic Review Software, 2019, Veritas) to manage the screening process, and duplicates were removed. Two reviewers (OW and ES) independently screened titles and abstracts of the search results. Where consensus was not reached, a third and more senior researcher (YP) was consulted. For studies and reviews that met the inclusion criteria, the full text was retrieved and screened by two researchers (OW and YP). Discrepancies were resolved by discussions between the two researchers.

### Data extraction

One author (OW) extracted relevant information from the included studies based on the Cochrane Consumers and Communication Review Group’s data extraction template [16]. Information extracted from each study included: study details (country, setting, date published), first author, study methodology (study design, recruitment, groups), participant characteristics (sex, age, MS phenotype, duration of disease, EDSS or PDDS score), study characteristics (intervention description, comparator/control description), description of primary and secondary outcomes (QOL or disability measurement tool used), results and a summary of the conclusions. Corresponding authors of studies with missing data were contacted via email to obtain additional information to be used in the synthesis of the results. No responses were obtained.

The effect measures of randomised control trials (RCT) included continuous outcome measures for both QOL and disability variables, which were summarised as the mean difference ($\pm$ standard deviation) from pre to post intervention, unless otherwise stated. For observational study designs, the relative risk was reported where available from the included studies.

### Data synthesis

The authors (OW, YP) identified what self-management regimen was being explored in each study and categorised it into one of five dimensions based on MS Australia’s Modifiable Lifestyle Factors Guidance document  [21]. This included: (1) physical activity, (2) diet, (3) fatigue management, (4) coping, depression, stress, and emotional management, and (5) symptom and medical management. Some interventions had multiple components (i.e. diet, exercise, and energy conservation); therefore, these interventions were categorised into a sixth dimension of (6) lifestyle and wellbeing programs. Individual studies were qualitatively synthesised under each self-management dimension to identify the findings related to participant outcomes. The outcomes are reported quantitatively in the final data summary table. The primary outcome measure was QOL (including their respective domains), and the secondary outcomes were measures of disability such as EDSS, relapse rate, and number and volume of T1 and/or T2 weighted brain lesions. Statistical significance was set at *P* < 0.05.

A meta-analysis was performed when there were two or more studies under the same self-management dimension that used the same disability measure or QOL domain with compatible summary measures (i.e. mean change (SD)), using Review Manager (RevMan) (Mac OS X, Version 5.4, The Cochrane Collaboration, 2020). Summary estimates were reported for studies of randomised controlled trials only, as the heterogeneity of comparing different study designs may increase the complexity of the analyses and impact the interrelation of quantitative outcomes [16, 28].

An inverse variance with random effects model was used because of the heterogeneity among participants in terms of MS disability and population characteristics [29]. The RevMan Calculator was used to calculate the standard deviation from the 95% confidence intervals and reported changes in means [30]. The *I*^2^ statistic was used to quantify the proportion of total variation attributable to between-study heterogeneity. Heterogeneity was categorised according to the Cochrane guidelines: (1) *I*^2^ = 0–40%: low heterogeneity; (2) *I*^2^ = 30–60%: moderate heterogeneity; (3) *I*^2^ = 50–90%, substantial heterogeneity; and (4) *I*^2^ = 75–100%: considerable heterogeneity [16].

### Risk of bias assessment

The risk of bias (RoB) of each study was appraised according to the study design. Version 2 of the Cochrane RoB tool, as recommended in the Cochrane Handbook for Systematic Reviews of Interventions [16], was used to assess RoB in each RCT. The studies were appraised according to the RoB arising from (1) the randomisation process, (2) deviations from the intended interventions (effect of assignment to intervention), (3) missing outcome data, (4) measurements of the outcome, and (5) selection of the reported result. A proposed RoB judgment for each domain was generated using an algorithm [31] and each study was assessed as having low risk, some concerns, or high RoB.

The RoB in non-randomised studies of interventions was assessed using the ROBINS-I tool [32]. This tool focuses on seven domains of bias including: bias due to confounding, selection of participants into the study, classification of interventions, deviations from intended interventions, missing data, outcome measurements and selection of the reported results. Risk levels of low, moderate, serious, critical, or insufficient information were assigned to each of these domains.

The RoB in case–control and cohort studies was assessed according to the Newcastle Ottawa Quality Assessment Scale (NOS) [33]. This scale was adapted from the NOS for cohort studies to provide RoB assessment of cross-sectional studies [34]. A star system was used to appraise each study under the following domains: (1) the selection of study groups, (2) the comparability of the study groups, and (3) ability to identify the exposure or outcome of interest. Each study was awarded one star for each criterion under the selection and exposure/outcome categories, and a maximum of two stars for the comparability category. Therefore, cohort/case–control studies with 7–8, 5–6, 4 and 0–3 stars were identified as very good, good, satisfactory, or unsatisfactory, respectively. Cross-sectional studies with 5, 4, 3 or 0–2 stars were identified as very good, good, satisfactory, and unsatisfactory, respectively [35].

### Certainty assessment

The certainty of the body of evidence of the quantitative synthesis was assessed using the Grading of Recommendations, Assessment, Development and Evaluation (GRADE) criteria [36]. The certainty of the body of evidence considered within-study risk of bias, precision and consistency of effect estimates, directness of evidence and risk of publication bias. An overall GRADE was determined as one of four levels of evidence: high, moderate, low and very low, by the two researchers (OW, YP). Randomised controlled trials begin with a high level of evidence. Factors that may increase or decrease the quality level of a body of evidence have been described elsewhere [36].

## Results

### Study selection

**Fig. 1:**
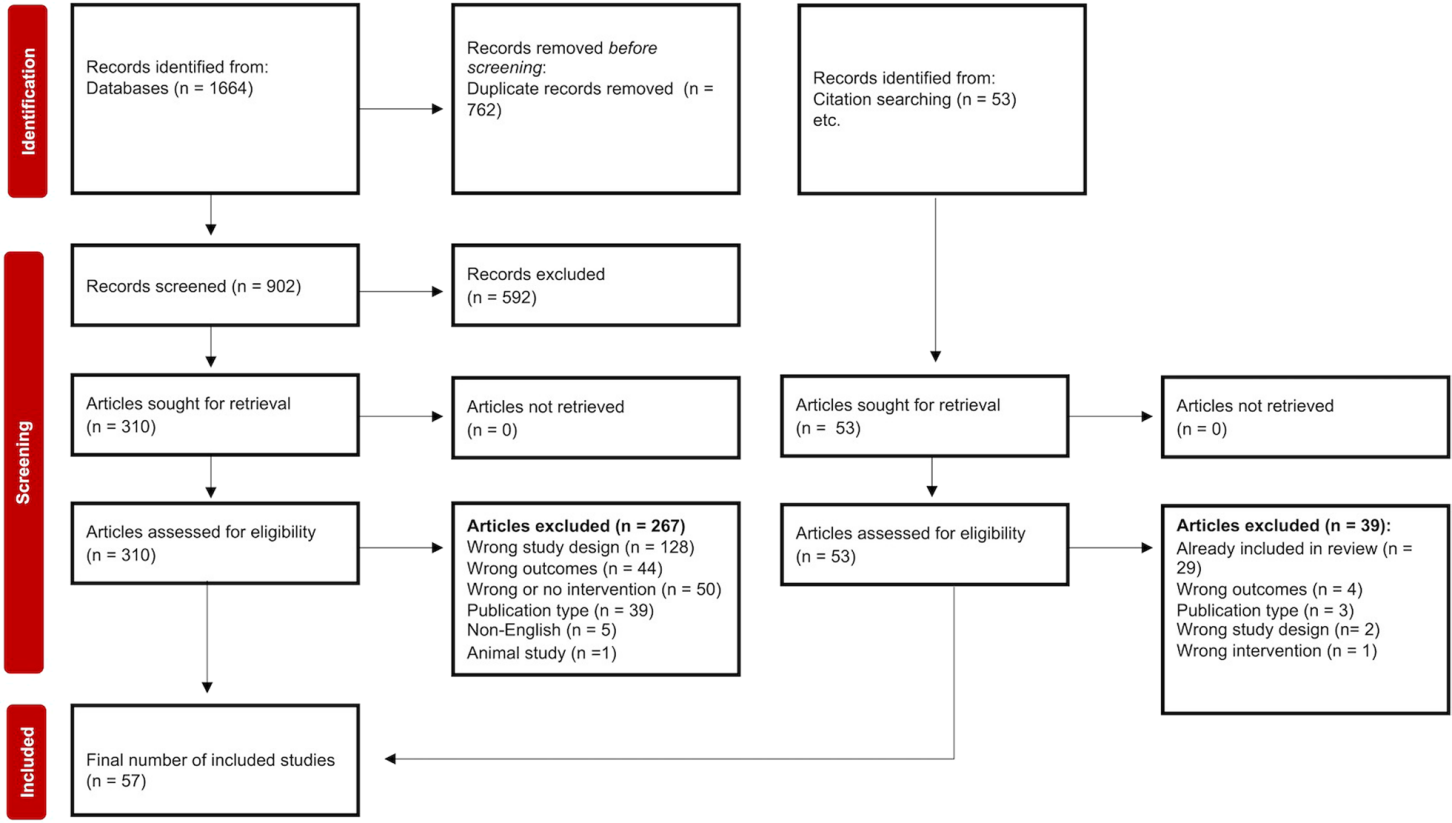
PRISMA flow chart of study selection

A total of 1664 references were identified from the database search, and an additional 14 references were obtained from manual searches. After removing the duplicates (*n* = 762), 902 references were retained. The full texts of 310 references were screened, and 43 studies met the inclusion criteria. Therefore, 57 studies were included in this systematic review [37–93]. the full selection process is presented in Fig. 1.

### Study characteristics

The included studies were published between 1996 and 2021, with 70% of studies published after 2010 and 33% of studies published after 2016. The highest proportion (35%) of included studies were conducted in the USA (*n* = 20) [40, 41, 47–49, 51–53, 59, 60, 63, 74, 81, 83–86, 88, 91, 93] followed by 28% in Europe (*n* = 16; Belgium, Italy, Netherlands, Slovenia, Denmark, Germany, Turkey, Finland, Norway and Switzerland) [39, 42, 43, 45, 46, 50, 55, 61, 62, 64–66, 68, 71, 73, 77], 16% in Asia (*n* = 9; Iran and India) [55, 57, 58, 69, 70, 72, 75, 82, 92] and 9% in both Australia (*n* = 5) [55, 56, 78–80] and UK (*n* = 5) [38, 67, 76, 89, 90]. In total, 70% (*n* = 40) of included studies were of experimental study designs of which 85% (*n* = 34) were RCT's [38, 43–46, 50, 51, 53–56, 59, 60–63, 66–76, 83, 84, 86, 88–91]. The remaining 30% of studies were of an observational study design including case–control (*n* = 8) [40, 41, 52, 65, 71, 81, 92, 93], cohort (*n* = 7) [37, 39, 64, 78–80, 87] and cross sectional analysis (*n* = 2) [47, 48] studies. The duration of the studies ranged from one week to five years. The full data summary is presented in Table 1.

**Table 1:** Results of included studies (n = 57) examining lifestyle management on QOL and disability, characterised according to self-management dimension

Author, year (country)	Study design	Study duration	Sample size (attrition)	Population characteristics, EDSS	RRMS	PPMS	SPMS	Mean disease duration, yrs	Intervention	Control	Self-management dimension	Outcomes (Measure)	Results	Key findings
Beatus et al. [37](USA)	Observational study	1 week	N = 41	Female 34, Male 7, Age 45 (8.54) EDSS unknown.				14.8 (8.38)	One-week summer retreat to encourage physical activity, art and social interaction.	NA	Physical activity	QOL (MSQOL-54)	No significant change between pre and post test on the physical component of QOL (P=0.214).A significance difference when comparing pre and post test measures on the mental portion of QOL (P=0.035).	Retreat had no positive effects on physical QOL, however did increase the mental perception of QOL in subjects with MS.
Carter et al. [38] (UK)	RCT	12 months	N = 120 (83%)	**Intervention**Female 43, Male 17, Age 46 (8.4) EDSS 3.8 (1.5) **Control:**Female 43, Male 17, Age 45.7 (9.1)EDSS 3.8 (1.5)	✓ N = 98	✓ N = 4	✓ N = 18	**Intervention:** 9.2 (7.9) **Control:** 8.4 (7.4)	12- week EXMES + usual care including: aerobic, strength, resistance training incorporating self directed exercise at home.	Usual care only	Physical activity	QOL (MSQOL-54) Disability (EDSS)	Difference in mean QOL change at 3, 9 months respectively:**Intervention:***Physical health component* 59.7 (20.6)*, 54.1 (21.7)*Mental health component*65.5 (20.2)*, 65.9 (21.0)**Control***Physical health component* 52.5 (21.4), 53.3 (21.1)*Mental health component*60.8 (20.0), 63.8 (24.1)*Significant change, P<0.05.There were no significant change in EDSS between in the intervention or control in 3 or 9 months post treatment (P>0.05).	Intervention could be an effective way to implement rehabilitation into a health care setting.
D'Hooghe et al. [39](Belgium)	Observational cohort study	10 months	N = 9 (100%)	Female 6, Male 3, Age 42 (23-40)^a^ EDSS 3 (1-4)^a^	✓ üN = 9			9 (3-24)^a^	5 day expedition to Machu Picchu (45.5km walk).(Fitness training 5 months prior + Follow up for 4 months post expedition).	NA	Physical activity	Walking ability (ESES)	ESES increased by 1 (36 – 37) within 8 months (P>0.05).The relapse rate during the study period did not differ from the relapse rate in the year prior.	Fluctuations were observed in self- reported disease steps
Fasczewski et al. [40](USA)	Mixed methods		N = 15	Female 11, Male 1 Age 43.5 (± 10.03)	✓ üN = 15			7 ± (± 4.34)	Qualitative interviews lasting 20- 60 mins were used to explore physical activity motivation and benefits. The transcribed interviews were coded into clusters of meaning.	NA	Physical activity	QOL(QOL survey)	**QOL measures:** Emotional: 19.56 (± 3.24) Physical: 20.34 (± 3.52)Two main themes emerged of how PA and QOL relate: (1) Physical fitness/strength (2) QOL/mental wellness/happiness.	Motivation to participate in long term PA reportedly increases QOL.
Fasczewski et al. [41](US)	Mixed methods	6 month	N = 16	Female 14, Male 2, Age 55.1 (± 9.93) PDDS 2.9 (± 1.79)	✓ N = 12	✓ N = 2	✓ N = 1	14.7 (± 11.72)	7x 90min medical therapeutic yoga sessions + education + relaxation therapy. A follow up structured interview used to assess retention of physical activity behaviours.	None	Physical activity (Medical therapeutic yoga therapy)	QOL(QOL-3) Disability (PDDS)	Only emotional QOL (P= 0.019) significantly improved between pre- and post-test measures. Greatest motivator for exercise was an improvement in QOL and functioning.	Intervention only increased emotional QOL.
Feys et al. [42](Belgium)	Uncontrolled interventional	10 months	N = 57 (74%)	**Group 1:** Female 22, Male 2, Age 37 (± 10) PDDS 0.9 (± 0.3) **Group 2:** Female 11 Male 7, Age 50 (± 11) PDDS 3.2 (± 1.3)	✓ N = 33	✓ N = 4	✓ N = 5	**Group 1:**7 (± 7) **Group 2**:12 (± 8)	Single education day followed by 3x 45 min practical sessions of different sports.Subgroups of disability level (Authors hypothesised that effects on education on physical activity could be greater in persons that have little physical restrictions).	NA	Physical activity	QOL(SF-36)	**Group 1 change at 6 months for:**Physical QOL: 0.21(6)Mental QOL: 0.6(7.7) **Group 2 change at 6 months for:** Physical QOL: -0.24(± 7) Mental QOL: -0.7(± 9.1) No significant change in QOL domains; Physical health (P=0.29) and Mental health (P=0.75) were found between groups or over time.	No significant difference was found between groups.
Flachenecker et al. [43](Germany)	RCT	6 months	N = 84 (76%)	**Intervention**Female 22, Male 9, Age 47.6 (± 9.2) EDSS 4.3 (IQR 1.5)^b^ **Control:**Female 17, Male 13, Age 46.4 (± 12.2) EDSS 4 (IQR 3)^b^	✓ N = 39			**Intervention:** 13.4 (± 7.9) **Control:** 9 (± 7.5)	Goal-oriented, multimodal inpatient rehabilitation program after discharge that promoted exercise.	Usual care	Physical activity	QOL (MSIS-29)	Improvements in HRQOL in intervention were sustained for up to 6 months (p < 0.001), whereas HRQOL in the control group returned to baseline scores at 3 months.Similar results for physical and psychological subscales of the MSIS-29.	With the reduction of fatigue, HRQoL increased.
Khan et al. [44](Australia)	RCT	12 month	N = 101 (98%)	**Intervention**Female 40, Male, 21 Age 49.7 (± 8.96) EDSS 0-3: 8, 3.5-6: 36, 6.5+: 17 **Control: **Female 32, Male 8, Age 51.2 (± 9.51) EDSS 0-3: 11, 3.5-6: 23, 6.5+: 6	✓ N = 31	✓ N = 14	✓ N = 56	**Intervention:** 10.52 (± 6.61) **Control:** 9.7(± 8.11)	Acute neurological and intensive MD rehabilitation (5 days x 3h therapy sessions a week). Intervention offered education, health promotion, bladder retraining and mobilisation.	Waitlist	Physical activity (Exercise)	QOL (GHQ-28)	Mean difference between intervention and control for GHQ subscales: Anxiety: -0.055 (CI: -1.85 – 1.74) Depression: 0.212 (CI: -1.32 to 1.74) Somatic: -1.25 (CI: -3.3 to 0.79) Social: -0.68 (CI: -2.91 to 0.83)	No significant effect of intervention on QOL. Therefore further exploration is required.
Kjolhede et al. [45](Denmark)	RCT	6 months	N = 35 (83%)	Age 43 (± 8) EDSS 2.9 (2-4)^a^	✓ N = 35			7 (± 7)	Progressive resistance training for 24 weeks, completed twice a week. Sessions consisted of four lower and two upper body exercises.	Habitual lifestyle. Followed interventiona after 24 weeks.	Physical activity (Resistance training)	Disability (EDSS, MRI)	Non- significant decrease in EDSS from baseline to T48 (P=0.75). All participants had a mean increase of 0.40 (CI: 0.001 - 0.558) in the number of lesions from T0 to T48 (p<0.01). No interaction was observed for T2 lesion volume.	Possible restorative effect of resistance training on brain structures, but the interpretation should be cautious.
Langeskov- Christensen et al. [46](Denmark)	RCT	6 months	N = 86 (73%)	**Intervention**Female 26, Male 17, Age 44 (± 9.5), EDSS 2.7 (± 1.4) **Control:**Female 26, Male 17, Age 45.6 (± 9.3) EDSS 2.8 (± 1.6)	✓ N= 75	✓ N= 6	✓ N= 5	**Intervention:** 10.9 (± 7.9) **Control:** 8.6 (± 6.0)	Twice weekly-supervised physical aerobic exercise followed by self guided physical activity.	Habitual lifestyle followed by supervised physical aerobic exericse.	Physical activity (aerobic exercise)	QOL (MSIS-29) Disability(T2 lesion count & load, relapse rate)	**Between- group change after 24 weeks:** Physical QOL: -1.6 (-6.6 to 3.5) Mental QOL: -3.4 (-9. to 2.9) T2 lesion count: 0.92 (-1.09 to 2.93) T2 lesion load: -0.16 (-0.73 to 0.40) Relapse rate: Intervention; 0 (0.0-0.07) *Control; 0.45 (0.28-0.61)*Significantly different	Intervention failed to affect QOL
Motl et al. [47] (USA)	Prospective cross sectional		N = 196	Female 173, Male 23, Age 46.1 (± 9.8)	✓ N = 174	✓ N = 3	✓ N = 19	9 (± 7.1)	Pedometer and accelerometer during waking hours for 7 days + A battery of questionnaires on the 8th day.	NA	Physical activity	QOL(SWLS)	The direct path between physical activity and QOL was non significant (.	Those with MS who were more physically active had greater self-efficacy and better functional capacity, which, in turn, was associated with greater QOL.
Motl et al. [48](USA)	Cross sectional	6 months	N = 292 (95%)	Female 245, Male 47, Age 48 years (10.3) EDSS Unknown	✓ N = 246	✓ N = 12	✓ N = 34	10.3 (± 7.9)	Accelerometer during waking hours for 7 days & repeated at 6 months.	No accelerometer	Physical activity	QOL: (LMSQOL)Disability: (PDDS)	Association between physical activity and QOL (P= 0.73). Indicators of physical activity and QOL (LMSQOL λ = 0.84) were statistically significant. Those who were more physically active reported higher QOL.	Indirect relationship between changes in physical activity and QoL over time, based on fatigue, pain, social support and self-efficacy.
Motl et al. [49] (US)	Panel study	6 months	N = 269 (98%)	Female 233, Male 46, Age 45.9 (9.6) PDSS 2 (0-6)^a^	✓ N = 233			8.8 (± 7)	Association between changes in physical activity and walking impairment. Frequency and intensity of participants exercise levels were recorded.	NA	Physical activity	Disability (PDDS)	**Mean change:**PDDS: -0.1 (1.0) Insignificant Significant path coefficients between baseline physical activity and walking impairment (P=0.0001) and between follow-up physical activity and walking impairment (P=0.01).	Results support change in physical activity as a determinant of walking impairments over time.
Mutluay et al. [50](Turkey)	RCT	6 weeks	N = 40	**Intervention**Female 8, Male 12, Age 40.3 (± 6) EDSS 4.85 (± 1.3) **Control: ** Female 8, Male 12, Age 38.1 (± 7) EDSS 4.18 (± 1.7)	✓ N = 12	✓ N = 8	✓ N = 20	**Intervention:** 9.8 (± 5.6) **Control:** 9 (± 4.6)	Home training programme, monitored by the physiotherapist by recalls to hospital for checking compliance with the assigned exercise schedule.	No exercises assigned.	Physical activity (breathing enhanced upper extremity exercise)	Disability (EDDS)	**Mean change:**Intervention: -0.23 (± 0.44)Control: 0.08 (± 0.37)Not significant (P > 0.05).	Intervention had no effect on EDSS.
Petjan et al. [51](Utah)	RCT	15 weeks	N = 54 (85%)	**Intervention**Female 15, Male 6, Age 41.1 (± 2) EDSS 3.8 (± 0.3) **Control:**Female 16, Male 9, Age 39 (± 1.7) EDSS 2.9 (± 0.3)				**Intervention:** 9.3 (± 1.6) **Control:** 6.2 (± 1.1)	15-week aerobic training program to improve measures of physical fitness.	No exercise	Physical activity	Disability (EDSS)	**Mean change:Intervention: **EDSS 3.8 (± 0.3) to 3.7 (± 0.3) **Control:**EDSS 2.9 (± 0.3) to 2.8 (± 0.3)Insignificant change P < 0.05	Intervention had no effect on EDSS.
Pilutti et al. [52](USA)	RCT	6 months	N = 82 (92%)	**Intervention:**Female 30, Male 11, Age 48.4 (IQR 9.1)^b^PDDS 2 (IQR 4)^b^ **Control:**Female 32, Male 9, Age 49.5 (IQR 9.2)^b^PDDS 3 (IQR 3) ^b^	✓ N = 65	✓ N = 7	✓ N = 10	**Intervention:** 10.6 (IQR 7.1)^b^ **Control:** 13.0 (IQR 9.1)^b^	Behavioural intervention that focused on increasing lifestyle physical activity through coaching sessions + a battery of questionnaires at the 6 month period.	Did not receive intervention	Physical activity	QOL(MSIS-29) Disability (PDDS)	No significant difference between groups on the physical and psychological QOL domains post intervention (P = 0.06 & P = 0.11 respectively). Mean difference between pre- post intervention not reported.	Lifestyle physical activity might be effective in supervised exercise training for improving HRQOL in MS patients.
Oken et al. [53](Portland)	RCT	6 months	N = 69 (83%)	**Exercise:**Female 13, Male 2, Age 48.8 (± 10.4) EDSS 2.9(± 1.7) **Yoga:**Female 20 Male 2, Age 49.8 (± 7.4) EDSS 3.2 (± 1.7) **Control:**Female 20 Male 0, Age 48.4 (± 9.8) EDSS 3.1(± 2.1)					90 min yoga classes once per week. With emphasis on breathing for concentration and relaxationORAerobic exercise + stretching	Waitlist	Physical activity (Yoga)	QOL (SF-36)	**Mean change:Exercise:**Physical QOL: 76.7 (± 25.8) to 61.7 (± 41.0) Emotional QOL: 82.2 (± 27.8) to 88.9 (± 30) **Yoga:**Physical QOL: 50 (44) to 48.8 (39.1)Emotional QOL: 72.4 (± 24) to 64.9 (± 17.9) **Waitlist:**Physical QOL: 40.3 (± 37.5) to 52.8 (± 43.6) Emotional QOL: 72.2 (± 43.2) to 72.2 (± 36.6)	Yoga intervention only improved the energy and Fatigue (Vitality) dimensions.
Sangelaji et al. [54](Iran)	RCT	14 months	N = 61 (90%)	**Intervention:**Female 24, Male 15, Age 33.05 (± 7.68) **Control:**Female 15, Male 7 Age 32.05 (± 6.35)					10 weeks (3x 60-90 min sessions per week) of combined exercise classes.	Avoided plannedphysical or rehabilitation activity for 10 weeks.	Physical activity (exercise therapy)	QOL specific for MS Disability (EDSS)	**Mean change at time 1:**Mental QOL 16.36 (*Physical QOL 12.17 (*EDSS -0.13 ( **Time 2:** Mental QOL 2.82 (Physical QOL -1.27 (EDSS -0.15 ( **Time 3:**Mental QOL 13.54 (*Physical QOL 10.90(*EDSS -0.28 (*P<0.05.	Rehabilitation can be important in keeping MS patients capable of doing their daily activities.
Savsek et al. [55](Slovenia)	Exploratory RCT	12 weeks	N = 28	**Intervention:**Female 11, Male 3, Age 39.7 (± 6.7) EDSS 2.5 (1.0-6.5)^a^ **Control:**Female 12, Male 2, Age 42.3 (± 5.7) EDSS 3 (1.0-6.0)^a^	✓ N=28			Intervention: 8.4 (± 6.1) Control: 14.8 (± 4.5)	60min aerobic exercise twice weekly. Participants followed prescribed exercise intensity and exercise. All participants were taking Fingolimod.	Normal daily routine	Physical activity (Aerobic exercise)	QOL (MSQOL-54) Disability (MRI- T2 lesion, EDSS)	**Mean difference between groups at 3 month follow up: ** EDSS: -0.31 (-0.84 to 0.22) Physical health composite QOL: -2.38 (-10.74 to 5.98) Mental health composite QOL: 1.47 (-8.13 to 11.07). Intervention was strongly associated with a decrease in lesion count: (T2 lesion count change: -0.13 (-1.90 to 1.64)).	Positive effect of intervention on the preservation of several regional brain volumes, possibly indicating a slowing of the neurodegenerative process in pwMS
Sutherland et al. [56] (Australia)	RCT (	10 weeks	N = 22	**Intervention**Female 6, Male 5, Age 47.18 (± 4.75) **Control:**Female 6 Male 5 Age 45.45 (± 5.05)				**Intervention:** 7 (± 5.59) **Control:** 6.18 (± 3.63)	Water and land aerobics 3x 45min weekly for 10 weeks.	No special activity	Aerobic exercise	QOL (MSQOL-54)	**Mean change:Intervention:**Physical: 15.2 (± 4.6) to 17.1 (± 4.4) Emotional: 19.6 (± 4.2) to 22.4 (± 4.1) **Control:**Physical: 15.4 (± 4.0) to 15.9 (± 4.0) Emotional: 20.7 (5.1) to 20.8 (6.7) 8 out of 11 subscales of QOL, showed a significant improvement (P<0.05)	Aerobic exercise can alter HRQOL for people with MS.
Vasudevan et al. [57] (India)	Single group pre/ post experimental	4 months	N = 10	Female 7, Male 3, Age range 31- 52					12 private customized yoga sessions and three group sessions, conducted by yoga therapists.		Physical activity (Yoga)	QOL (MSQOL-54)	**QOL domain: **^b^Physical composite: 72.1 (IQR 57 - 80) – 78.9 (69 - 81) Mental composite: 73 (65 - 85) to 85.3 (84 - 88) Overall: 73.4 (64 – 83) to 79.2 (70 – 87)Not significant (P>0.05).	Yoga may be encouraged in addition to medical management for better QOL, which includes social and cognitive function.
Abolghasemi et al. [58](Tehran city)	Experimental pre test/ post test with control		N = 32	**Intervention**Female 7, Male 9, Age 31.75 ((8.25) **Control:**Female 9 Male 7, Age 32.5 (90.58)					12x 75 min supportive-expressive therapy sessions. At the twelfth session, subjects were asked to answer questionnaire’s on quality of life again.	Followed the medicaltreatments	Coping, depression, stress and emotional management	QOL (WHOQOL-B)	**Mean QOL pretest VS posttest:**Intervention: 35.47 (6.76) VS 54.06 (7.05) *Control: 40.65 (14.66) VS 42.29 (12.48)**Significant difference between pre and post-intervention scores in both groups (P<0.01). The effect of supportive-expressive therapy on enhancing quality of life was 0.418.	Intervention is effective for enhancing the QOL MS patients.
Besharatet al. [59] (US)	RCT	3 months	N = 24	**Intervention**Female 12, Male 0, Age 35 (7.45) EDSS 2.3 **Control:**Female 12, Male 0, Age 30 (± 4.1) EDSS 2.7				Intervention: 2.8 Control: 3.25	Mindfulness based stress reduction program. Training sessions involved mindfulness awareness during yoga/ stressful situations/ social interactions, body scan meditation.	Standard medical care for MS	Coping, depression, stress and emotional management (Stress management)	QOL(SF- 36)Disability (EDSS)	Mean QOL:Intervention -3.1 (± 4.74)*Control 2.51 (± 2.28)*Significant increment in quality of life.	Training in mindfulness may offer MS patients a self-management of symptoms that enhances QOL.
Ehde et al. [60](USA)	RCT	12 months	N = 163	**Intervention**Female 67, Male 8, Age 51 (± 10.1) EDSS <4: 194.5 – 6.5: 46>7: 10 **Control: ** Female 75, Male 13, Age 53.2 (± 10) EDSS <4: 234.5 – 6.5: 55>7: 10	✓ N = 91	✓ N = 72		I**ntervention:**<5: 215-9: 1710-19: 29>20: 8 **Control:**<5: 215-9: 2510-19: 26>20:16	Individual telephone delivered self management intervention including evidenced based cognitive behavioural and positive psychology strategies	Education in MS care	Coping, depression, stress and emotional management	QOL (SF-8)	Physical HRQOL significantly improved (CI:-5.51 to -1.45) post treatment, favouring the self management intervention.This was maintained 6 months post intervention (-5.1o to -0.75, however not 12 months post. P<0.05.Mental HRQOL significantly improved post treatment, at 6 and 12 months in both intervention and control (P<0.05).	Validates telephone as a simple but effective method for improving aspects of QOL in the short term.
Grossman et al. [61](Switzerland)	RCT	8 months	N = 150	**Intervention**Female 59, Male 19, Age 45.93 (± 10) EDSS 3.03 (± 1.12) **Control:**Female 60, Male 14, Age 48.68 (± 10.58) EDSS 2.98 (± 0.83)	✓ N = 123		✓ N = 27	**Intervention:** 7.74 (± 0.9) **Control:** 9.71 (± 0.88)	Mindfulness based intervention (8-week 2.5h class program), based on: perception, acceptance of health changes, sense of control.	Regular medical care	Coping, depression, stress and emotional management (Mindfulness)	QOL (HAQUAMS)	**Mean change QOL:Intervention**Pre/ post intervention: 0.18 (0.09 to 0.27)*6 month f/ u: 0.13 (0.00 to 0.25)* **Control:**Pre/ post intervention: -0.09 (-0.20 to 0.01)6 month f/ u: -0.05 (-0.16 to 0.07) *Significant difference in HAQUAMS QOL, P<0.05.	MBI may improve HRQOL for at least 8 months among mild to moderately severely impaired patients with MS.
Graziano 2014 [62] (Italy)	RCT	6 months	N = 82 (66%)	**Intervention**Female 27, Male 14, Age 42.3 (8.5) **Control:**Female 24, Male 17, Age 38.3 (10.1) EDSS Unknown	✓ N= 77	✓ N= 2	✓ N= 3	**Intervention:** 8.6 (5.2) **Control:** 7.2 (5.3)	Four group based cognitive behavioural therapy sessions (2hr) over two months + a 6-month follow up + home relaxation tasks and self-practice of exercise.	Informative sessions about stem cells, CAM and nourishment.	Coping, depression, stress and emotional management (Depression)	QOL(MSQOL-54)	**QOLIntervention VS control**Pretreatment: 13.39 (4.39) VS 12.43 (4.54)Posttreatment: 14.24 (3.62) VS 13.71 (4.00)6- months post: 14.96 (4.28)* VS 11.95 (5.40)* *Mean difference between groups at 6-month follow-up was significant (P = 0.034).	QOL increased over time in the intervention and decreased in control, which was found to be significant (P = 0.042).
Hart et al. [63](USA)	RCT	16 weeks	N = 60 (82.2%)	Female 44, Male 16, Age 44.8 (± 10.3)	✓ N = 60			8.3 (3 months – 31.2 years)^c^	Individual, weekly cognitive behavioural therapyOR Supportive expressive group psychotherapyOR	Sertraline (medication).	Coping, depression, stress and emotional management	QOL (MSQOL-54) Disability(T25FW)	**Mean QOL pre and post treatment:**Physical 45.3 (15.6) VS 52.8 (16.0)*Mental 39.7 (14.9) VS 60.0 (21.1)**Significant improvements over time in the physical health composite scale, P<0.001.Change in depression scores significantly predicted post-treatment scores for the MSQOL total score, physical health composite scale, and the mental health composite scale (P<0.001).	Treatment for depression was related to better QOL in MS patients.
Jongen et al. [64](Netherlands)	Observational cohort study	6 months	N = 94 (47 PwMS + support partners (93%)	**RRMS group 1:**Female 16, Male 4, Age 42.7 (± 10.1) EDSS 3.1 (± 1.2) **PPMS group 1:**Female 19, Male 5, Age 48.7 (± 7.6) EDSS 5.5 (± 1.4)	✓ N = 20	✓ N = 2	✓ N = 22	**Group 1:**8.4 (6.9) **Group 2:**17.5 (8.6)	Social Cognitive Can Do Program (SCDP) with participants and partners involved group sessions, consultations, theatre evening and joint activities.	NA	Coping, depression, stress and emotional management (stress management)	QOL (MSQOL-54) Disability (EDSS)	**Mean (SEM) % QOL change:RRMS group 1:**Physical 1 month: 6.0% (5)Physical 3 months: 6.3% (5.9)Physical 6 months: 12.7% (4.8)* Mental 1 month: 21.4% (8.8)*Mental 3 months: 22.3% (10.7)*Mental 6 months: 22.3% (8.7)* **PPMS group 2:**Physical 1 month: 5.3% (8.2)Physical 3 months: 8.4% (10.7)Physical 6 months: 7.8% (8.0) PPMS group 2: Mental 1 month: 11.5% (5.4)Mental 3 months: 5.5% (5.4)Mental 6 months: 3.2% (7.9) *Significant result, P<0.05.	6 months post SCDP; PwMS with a RR course or low disability may experience an improved mental and physical HRQoL.
Jongen et al. [65](Netherlands)	Exploratory, uncontrolled	12 months	N = 60 (66.7%)	Female 25, Male 13 EDSS unknown	✓ N = 22	✓ N = 14			As above.	NA	Coping, depression, stress and emotional management (stress management)	QOL (MSQOL-54)	Twelve months post treatment, physical QOL improved by almost 15%, reaching significance (P=0.032). Mental QOL increased by 17%, however this change was not significant (P=0.087).	12 months post treatment, persons with RRMS showed improved physical HRQoL.
Jongen et al. [66](Netherlands)	RCT	3.5 years	N = 158 (76%)	**Intervention**Female 69, Male 10, Age 40 (± 8.7) EDSS 2.3 (± 1.03) **Control**: Female 70, Male 9, Age 40 (± 9.4) EDSS 2.3 (.13)	✓ N = 158			**Intervention:** 6.5 (± 5.6) **Control:** 6.5 (± 5.3)	As above.	Any care or treatments that were deemed necessary by caregivers.	Coping, depression, stress and emotional management (stress management)	QOL (MSQOL-54)	**Mean QOL change:Intervention VS control**Physical baseline: 53.2 (13.6) VS 54.7 (15.3)Physical 1 month: 55.3 (15.1) VS 64.1 (14.6)*Physical 3 months: 56.4 (14.3) VS 60.7 (17.1)Physical 6 months: 58.1 (14.0) VS 59.2 (17.2) Mental baseline: 63.4 (20.1) VS 60.9 (17.6)Mental 1 month: 64.1 (19.2) VS 69.1 (15.7)*Mental 3 months: 66.2 (20.2) VS 67.0 (16.2)Mental 6 months: 67.3 (18.2) VS 65.9 (17.8) *Significant result, P<0.05.	Intervention favoured CDT group at one month.
Lincoln et al. [67](UK)	RCT	8 months	N = 151 (85%)	**Intervention**Female , Male, Age 44.5 (± 11.1) **Control:**Female , Male,Age 47.5 (± 10.5)	✓ N = 103	✓ N = 15	✓ N = 30	**Intervention:** 9.2 (7.8) **Control:** 10.5 (8.0)	6x 2h group treatment sessions over 12 weeks. Sessionscovered topics including problem-solving,realistic goal setting and mental health and were taught strategies to reduce distress.	Received all otherrehabilitation routinely provided.	Coping, depression, stress and emotional management (stress management)	QOL(MSIS-29) Disability (Guy’s Neurological Disability Scale)	**QOL difference (95% CI)**Physical 4 months: 0.14 (12.3 to 0.8)*Physical 8 months: 0.20 (15.1 to 4.4.)* Psychological 4 months: 0.12 (11.8 to -0.6) Psychological 8 months: 0.20 (15.1 to 4.4)*Significant difference, P<0.05. There was a significant difference between intervention and control for psychological QOL (P=0.037), but not for physical QOL (p-0.149).	Significant difference in psychological impact of MS QOL.
Aivo et al. [68](Finland)	RCT	12 month	N = 30 (90%)	**Intervention**Female 9, Male 6, Age 37 (25-53)^a^ EDSS 2 (0-3.5)^a^ **Control:** Female 9, Male 6, Age 32 (22-47)^a^EDSS 2 (0-4)^a^				**Intervention:** 3 (0.6-15.2)^a^ **Control:** 1.5 (0.3-4.7)^a^	20mg of cholecalciferol / 20000 IU of vitamin D3 , administered as one capsule once a week	Placebo	Diet (Vitamin D supplementation)	Disability (EDSS, MRI)	**Mean difference in EDSS pre/ post intervention:Intervention:** -0.3 (0.6)**Control:** -0.1 (0.7)No significant change (P=0.27). Number of T1 lesions decreased in both groups (control; P = 0.018, vitamin D-treated group;P = 0.027). Number of new or enlarging T2 weighted lesions was greater in the placebo group (P = 0.132).	Intervention reduced T1 enhancing lesions and lesion volume growth.
Ashtari et al. [69](Iran)	RCT	3 months	N = 94	**Intervention**Female 37, Male 10, Age 31.4 (± 7.6) EDSS 1.7 (± 0.6) **Control:**Female 41, Male 6, Age 34.6 (± 10.1) EDSS 2 (± 0.9)	✓ N = 94			**Intervention:** 4.1(± 3.73) **Control:** 4.4 (± 3.9)	50,000 IU vitamin D3 every five days for 3 months+ Interferon-β (IFN-β) as main treatment.	Placebo + Interferon-β (IFN-β)	Diet (Vitamin D supplementation)	QOL (MSQOL-54) Disability (EDSS)	**Mean difference in QOL components after 3 months:Intervention:**Physical: 71.74 (Mental: 62.41 (*ControlPhysical: 69.55 (Mental: 60.99 (* *Significant difference after adjustment for age, sex, disease duration, and EDSS.	A positive change in mental QOL was reported by patients in intervention.
Bitarafanet al. [70](Iran)	RCT	12 month	N = 101 (92%)	**Intervention**Female 35, Male 12, Age 30.4 (6.9) EDSS 1.3 (± 0.97) **Control:**Female 34, Male 12, Age 32.3 (± 5.9) EDSS 1.4 (± 1.05)	✓ N = 101			**Intervention**: 4.3 **Control:** 5.3	25000IU Vit. A for six months followed by 10000IU/d Vit. A for another six months.	Placebo	Diet (Vitamin A supplementation)	Disability (Relapse rate, MRI brain scan)	**Mean difference in EDSS pre/post intervention:Intervention: **0.07 (0.23)**Control: **0.08 (0.23) No significant difference between groups for relapse rate, number of enhanced lesions, volume of T2 hyper intensive lesions, new T2 lesions (P>0.05).	Vitamin A supplementation does not affect disease activity, but may inhibit disease progression in MS patients.
Kampan et al.[71](Norway)	RCT	96 weeks	N = 71 (96%)	**Intervention**Female 24, Male 11, Age 40 (21-50)^c^ EDSS 2.5 (0-4.5)^c^ **Control:** Female 24, Male 9, Age 41 (26-50)^a^ EDSS 2 (0-4.5)^a^	✓ N=68			Intervention: 11 (1-27)^c^ Control: 10 (2-26)^c^	20,000IU vitamin D3 + 500 mg elemental calcium daily.	Placebo + calcium tablet	Diet (Vitamin D supplementation)	Disability (EDSS, ARR)	**Mean difference in EDSS pre/ post intervention:Intervention:** 0.16 (0.73)**Control:** 0.15 (0.71)No significant change (P=0.97).No significant change in ARR between groups after the intervention period (P=0.25).	ARR was not reduced and EDSS was unchanged in intervention group.
Kouchaki, E. et al. [72](Iran)	RCT	12 weeks	N = 60 (100%)	**Intervention**Female 25, Male 5, Age 33.8 (8.9) EDSS 2 (0-4.5)^a^ Control: Female 25, Male 5, Age 34.4 (9.2) EDSS 2.5 (0-4)^a^	✓ N = 60			**Intervention:** 4.3 (2.8) **Control:** 4.3 (2.9)	Probiotic capsule containing Lactoba- cillus acidophilus, Lactobacillus casei, Bifidobacterium bifidum and Lactobacillus fermentum.	Placebo	Diet (Probiotic supplementation)	QOL(GHQ-28) Disability (EDSS, relapse rate)	**Mean difference in EDSS pre/ post intervention:Intervention:** -0.3 (0.6)**Control:** 0.1 (0.3)Probiotic intake significantly improved EDSS (P= 0.003) and GHQ-28 scores (P=0.002).	Probiotic intake had favourable effects on EDSS.
Torkildsen et al. [73](Norway)	RCT	24 months	N = 92 (99%)	**Intervention**Female 30, Male 16, Age 38.8 (± 8.4) EDSS 1.94 (± 0.78) **Control:** Female 29, Male 16, Age 38.3 (± 8.4) EDSS 1.86 (± 0.86)	üN = 92			**Intervention:** 5.4 (5.4) **Control:** 5.8 years (5.9)	Oral omega- 3 fatty acid supplementcontaining EPA & DHA + 4iu/ gram tocopherol were added for antioxidative protection.After 6 months, all participants (intervention + control) received 44 μg of interferon beta-1a (Rebif ) 3 times per week for another 18 months.	Placebo	Diet (Omega- 3 supplementation)	Disability (MRI brain scans, relapse rate, EDSS)	No significant effect of intervention on number of new T1 weighted Gd enhanced lesions, at 6, 9 or 24 months (P = .09, P=0.10, P=0.17, respectively).No significant difference between the treatment groups regarding new T1-weighted hypointense lesions after 24 months of treatment (P = 0.40).No difference in EDSS scores between the treatment groups after 24 months (P=0.63).	No beneficial effects of omega-3 fatty acid supplementation on disease activity in MS.
Weinstock-Guttman et al. [74](NY)	RCT	12 months	N = 31 (87%)	**Intervention**Female 12, Male 2, Age 45.1 (± 7.7) EDSS 1.9 (± 0.6) **Control:**Female 11, Male 2, Age 39.9 (± 10) EDSS 2 (± 1.3)	✓ N = 31			**Intervention:** 6.9 (5.9) **Control:** 4.6 (3.5)	6x fish oil capsules per day containing 1 g FO (65% o-3; EPA 1.98 g and DHA 1.32 g/day)+ Very low fat diet intake, (<15%).+ 400 units of Vit. E+ 1x multivitamin tablet+ 500mg calcium per day.	Olive Oil group+ Low cholesterol diet (<30% TF)+ Vit E, multivitamin+ 500mg calcium supplement.	Diet(Fish oil supplementation + low fat diet)	QOL(SF-36) Disability (EDSS, relapse rate)	Significant change in physical composite scale (P=0.05) observed between intervention and control at 6 months.Worsening in EDSS scoers in the olive oil group (+0.0.35 EDSS points)Decrease in EDSS in the fish oil group (-0.07 EDSS points).Significant decrease in relapse rate when compared to 1 year prior to the study in both groups (P= 0.021, P=0.044).	Low fat diets have potential to improve the physical and emotional disease burden.Low fat diet + fish oil was more efficient at doing so.
Zandi- Esfahan, S. et al. [75](Iran)	RCT	12 months	N = 50 (82%)	**Intervention**Female 13, Male 12, Age 35.19 (± 9.97) **Control: ** Female 16, Male 9, Age 31.4 (8.41)	✓ N = 50				Fingolimod + Fish oil (180 mg (EPA), 120 mg (DHA), and excipient (glycerin, purified water, tocopherol, sunflower oil, and tita- nium dioxide)	Fingolimod + Placebo - capsules	Diet(Fish oil supplementation)	Disability (EDSS)	**Mean difference in EDSS pre/ post intervention:Intervention: **0.786 (1.04)**Control: **0.875 (0.67)Changes between two groups showed no significant difference (P= 0.747).	Fish oil had no role in improving patients’ disability progression. Decreased EDSS can be attributed to taking Fingolimod.
Ennis et al. [76](UK)	Single blinded RCT (II)	8 weeks	N = 62(32 intervention, 30 control) (98%)	**Intervention**Female 20, Male 11, Age 45(± 9) **Control:** Female 19, Male 11, Age 46 (± 8)	✓ N = 28	✓ N = 11	✓ N = 20	**Intervention:**7 (5) **Control:** 8 (6)	OPTIMISE program (3h weekly sessions) to provide people with the knowledge, skill and confidence to undertake health promoting activities.	Present level of care	Lifestyle & wellbeing (Exercise and physical activity, fatigue management, stress management, diet)	QOL(SF-36)	**Mean change in SF-36 domains pre/ post intervention:Intervention:**Role Physical: 23.29 (± 53.98) (Role Emotional: 15.0 (± 88.91) (**Control:**Role Physical: -3.33 (± 36.98) (Role Emotional: 8.87 (± 40.07) Physical domain, mental health and general health domain were statistically significant in the intervention compared to the control group P=0.03, P<0.01, P<0.01 respectively.	Increase in health promotion has a significant effect on QOL.
Feicke et al. [77](Germany)	Prospective quasi- experimental evaluation study	6 months	N = 81 (73%)	**Intervention:** Female 27, Male 4, Age 41.94 (± 11.7) **Control: ** Female 23, Male 10, Age 37.12 (± 7.83) EDSS unknown	✓ N = 32	✓ N = 2	✓ N = 2	**Intervention:** 0.97 (1.11) **Control:** 1.64 (1.45)	S.MS program which involved physical and emotional management strategies and support.	Received brochurewhich covered the same content as the training program.	Lifestyle & wellbeing (Disease management, support strategies, coping strategies)	QOL (HAQUAMS)	**Mean changes in HAQUAMS from pre intervention to six month follow up:Intervention**: 1.86 (± 0.55) to 1.84 (0.56)**Control:** 1.80 (± 0.52) to 2.00 (± 0.67) MS specific QOL decreased in control group and increased in the intervention from post intervention to 6 months follow up.	Compared to the control group, ‘‘S.MS’’ participation was associated with a significant, sustained improvement of self-management and disease-specific quality of life.
Hadgkiss et al. [78](Australia)	Longitudinal cohortstudy	5 year follow up	N = 387 attended retreat over 8 years (71%)	Female 227 Male 47, Age Unknown EDSS Unknown					Five day live in educational programme to assess the impact of modifiable lifestyle factors on MS outcomes.	NA	Lifestyle & wellbeing (Diet, exercise, stress management)	QOL (MSQOL-54)	**One year follow changes were significant in:**(P<0.001): role limitations- physical, emotional well- being, energy, health perceptions, cognitive function, health distress, overall QOL, change in health, physical and mental health composite), role limitations- emotional,(P=0.002): pain(P=0.001): social function **Five year follow up changes were significant in:**(P<0.001): Role limitations- physical, role limitations- emotional, emotional well- being, energy, health perceptions, health distress, overall QOL(P=0.004): Physical and mental health composite,(P=0.043), social function cognitive function(P=0.009): Change in health	Overall QOL improved by 11.3% (p<0.001).Overall improvement in quality of life of 19.5% (p<0.001).
Li, et al. [79](Australia)	Longitudinal cohortstudy	2.5 year follow up	N = 188 (58%)	Female 94, Male 15, Age Unknown EDSS Unknown					As above	NA	Lifestyle & wellbeing (Diet, exercise, stress management)	QOL (MSQOL-54)	**One year follow changes were significant in:**(P<0.001): role limitations- physical, health distress, overall QOL and mental health composite score. P=0.001: emotional wellbeing, energy, physical health composite score. P=0.019: health perceptions, P=0.04: cognitive function, P=0.012: sexual function, P=0.008: role limitations- emotional, P=0.002: pain, P=0.001: social function **At 2.5 year follow up changes were significant in:**P=0.015: emotional wellbeingP=0.044: energy,P=0.013: health perceptions,P=0.001: health distress,P=0.035: sexual function,P=0.031: Overall QOL,P=0.014: physical health composite, P=0.008: mental health composite	A significant improvement in short- and medium-term HRQOL for people with MS, 1 and 2.5 years after intervention.
Marck et al. [80](Australia)	Observational study	3 years	N=95 (82%)	Female 69, Male 26, Age 44 (± 10.5)EDSS unknown	✓ N = 68	✓ N = 9	✓ N = 18 (Benign/ other)	49.5% = <2yr19%= 2-5yr17.9%=6-10yr13.7%= >11	5 day group workshop delivered by health care professionals to develop self- management skills.	NA	Lifestyle & wellbeing (Diet, exercise, stress management)	QOL (MSQOL-54)	**Mean (95% CI) change:Mental health QOL:**1 year: 9.2 (5.8 to 12.6)*3 years: 8.0 (4.2 to 11.8)* **Physical health QOL:**1 year: 8.0 (5.2 to 10.8)*3 years: 8.7 (5.3 to 12.2)* Significant improvement P<0.05.	QOL maintained up to 3 years post intervention.
Ng et al. [81](Canada)		6 months	N = 129 (64%)	Female 99, Male 30, Age 49 (11) EDSS 3.5 (-9.5)^a^				4 (± 2)	Interdisciplinaryeducational wellness program, consisting of physical and psychological evaluations, lectures and workshops.	NA	Lifestyle & wellbeing (Symptom management, diet, exercise and physical activity, emotional management, goal setting, stress management, energy conservation)	QOL(SF-36)	Significant improvements in:Physical function at 1 month (p < 0.01) and 3 months (p = 0.04).Role physical subscale at 6 months (p = 0.02).Mental Health at 1 (p = 0.004) and 6 months (p = 0.01).No change after program participation in the remaining SF-36 categories; bodily pain, social function or role emotional (P>0.05).	Short-term wellness-program can result in significant positive changes in QOL for plwMS. These changes are stable up to at least 6 months.
Sahebalzamani et al. [82](Iran)	Quasi experimental	3 months	N = 53 (94%)					1-5 years: 25>5 years: 25	3- month self- care training program involving 6 x 50minute training sessions.	NA	Lifestyle & wellbeing (Symptom management, emotional management)	QOL (MSQOL-54)	**Mean difference pre VS post intervention:**Physical health: 12.86 (± 2.39) VS 12.9 (± 2.39)*Mental health: 10.06 (± 1.88) VS 10.08 (1.88)* *Significant improvement (P<0.05) + * For pain, fatigue, perceived health, overall health, marital satisfaction, psychological health, feeling of wellbeing.	Self-care training to be effective on physical, emotional and psychological domains of quality of life in patients with MS.
Stuifbergen et al. [83](USA)	RCT	8 months	N = 121 (93%)	Female 113 Male 0, Age 45.97 (± 10.09) EDSSUnknown	✓ N=62			10.76 (± 6.62)	8- week life style program (8x 90 min sessions)+ Bimonthly telephone calls for up to 3 months post program.	No intervention	Lifestyle & wellbeing (physical activity, diet, stress management, women's health)	QOL(SF-36)	**Mean QOL domain at baseline VS T4:Intervention:Role- physical: **42.9 (40.7) VS 46.9 (43.8)**Role- emotional: **63.1 (40.5) VS 76.2 (36.0)**Control:**Role- physical: 38.2 (38.1) VS 41.4 (42.0)Role- emotional: 67.8 (42.2) VS 65.5 (42.5) Bodily pain and mental health (P<0.05) were higher for the intervention group.Physical function, role- emotional, role-physical, bodily pain, social functioning, general health, mental health, vitality was significant (P<0.01).Stability in scores achieved by month 2	Intervention improved QOL domains: pain & mental health.
Finlayson et al. [84](USA)	RCT	6 months	N = 181 (80%)	Female 143, Male 38, Age 56 (9)PDDS 4 (2)	✓ N=95	✓ N=16	✓ N=39	15 (± 9)	6 -week, group based intervention. 70 min teleconference calls facilitated by an OT.	Waitlist	Fatigue management	QOL(SF-36) Disability (PDDS)	**Mean (95% CI) difference between intervention and control pre/post intervention in QOL domains:**Vitality: 6.99 (4.29 to 9.69)*Role emotion: 10.08 (4.13 to 16.04)*Mental health: 5.78 (3.89 to 7.67)*Social function: 7.95 (4.09, 11.82)*Role physical: 11.12 (6.22 to 16.02)*Physical function: 2.62 (0.52 to 4.71)*Significant difference P<0.05/8	Intervention was more effective than control for reducing fatigue impact and improving role- physical domain of QOL.
Mathiowetz et al. [85](USA)	Repeated measures clinical trial	19 weeks	N = 54	Female 36, Male 18, Age 50 (31-74)^c^	✓ N = 20			9.5 (1-34)^c^	6x 2h weekly sessions incorporating lectures, discussions, long- and short-term goal setting, activity stations, and homework activities to teach energy conservation principles.	6 weeks symptom management course followed by 6 week intervention course.	Fatigue management (energy conservation)	QOL(SF- 36)	**Mean difference week 1 VS week 19:**Role physical: 26.9 (29) VS 26.9 (34)*Role emotional: 49.4 (41) VS 60.9 (42)Vitality: 34.1 (18) VS 43.3 (20)*Social: 59.1 (24) VS 67.5 (22)*Mental health: 65.9 (17) VS 71.2 (16)**Significant improvement post intervention, P<0.001.	Intervention is effective in improving some aspects of QOL for individuals with MS.
Mathiowetz et al. [86](USA)	RCT	6 weeks	N = 169(77%)	Female 140, Male 29, Age 48.34 (8.44)	✓ N = 104	✓ N = 10	✓ N = 32	9.47 (± 7.44)	Energy conservation course. 2h classes that covered rest, communication techniques, body mechanics and prioritisation.	6 week delayed intervention.	Fatigue management	QOL(SF-36)	**Mean (95% CI) difference between intervention and control in QOL domains:**Role- Physical: 15.18 (0.78 to 29.57)*Role- emotional: 13.23 (-6.77 to 33.24)Vitality: 11.64 (5.48 to 17.79)*Mental health: 6.12 (0.01 to 12.24)** Significant improvement, P<0.05.	Intervention increased some aspects of quality of life (vitality, mental health, physical health).
Mulligan et al. [87] (NZ)	Observational cohort study	3 months	N = 23 (88%)	Female 23, Male 0, Age 48.96 (± 8.13) EDSS unknown	✓ N = 14	✓ N = 2	✓ N = 3	11.52 (± 9.95)	Minimise fatigue, maximise life program: 6 weeks, 2hr sessions/ wk.	NA	Fatigue management	QOL(SF-12)	Themes:(1) Achieving behaviour change to manage fatigue(2) Whole life effects	Minimise fatigue, maximise life positively affected lives of participants.
Plow et al. [88](Ohio)	RCT	6 months	N = 208 (78.4%)	Female 176, Male 32, Age 52.1 (± 8.4) PDDS:Mild: 34 Moderate: 41 Gait: 62 Early cane: 45 Late cane: 26	✓ N = 176	✓ N = 6	✓ N = 11	12.7 (± 8.6)	3 teleconference sessions+ 4 individually tailored phone calls + Fatigue course for energy conservation.	(1) PA only(2) CC Health information	Fatigue management	QOL(MSIS)	**Mean (95% CI) difference post test:PA- only VS CC:**Physical function: -6.03 (-12.90 to 0.85)Mental function: -3.27 (-10.18 to 3.63)**FM VS CC:**Physical function: -5.60 (-12.18 to 0.99)Mental function: -3.40 (-9.96 to 3.17)**FM VS PA- only:**Physical function: 0.43 (-6.26 to 7.12)Mental function: -0.12 (-6.82 to 6.58)	Intervention had no effect on improving QOL.Future studies can experiment with number of teleconference sessions.
Thomas et al. [89](UK)	RCT	18 months	N = 164 (89%)	**Intervention**Female 61, Male 23, Age 48 (10.2) **Control:** Female 58, Male 22, Age 50.1(± 9.1)	✓ N = 75	✓ N = 13	✓ N = 39	<1 year: 6, 1-5 years: 53,6-10 years: 32,11-15 years: 33, >16 years: 34	6x 90min sessions of the FACETS intervention plus current practice to assist	Current local practice alone	Fatigue management	QOL(MSIS-29 V.1, SF-36)	**Mean (95% CI) difference between intervention and control:MSIS-29: **1 year: 1.44 (-2.36 to 5.24)2 years: -1.56 (-6.45 to 3.34)No significant differences between the intervention and control for the MSIS-29 at follow up 1: P=0.46 OR follow up 2: P=0.53.	It may take longer for the intervention to impact on QOL. Therefore longer term follow up may be required.
Thomas et al. [90](UK)	RCT	1 year	N = 164 (80%)	**Intervention**Female 61, Male 23, Age 48 (± 10.2) PDDS <3: 184-5: 376>: 26 **Control:**Female 58, Male 22, Age 50.1 (± 9.1) PDDS <3: 154-5: 426>: 21	✓ N = 75	✓ N = 13	✓ N = 39	**Intervention:**<1: 61-5: 536-10: 3211-15: 33>16: 34	FACETS programme (6x 90 min sessions, weekly) for fatigue managmenet.	Received current local practice.	Fatigue management	QOL(MSIS-29, SF-36)	**Mean (95% CI) difference between intervention and control:Physical:**1 year: 1.39 (-2.87 to 5.65)2 years: -0.81 (-5.91 to 4.28)3 years: -4.74 (-9.4 to -0.08)* **Vitality:**1 year: 4.42 (-1.22 to 10.06)2 years: 6.38 (0.45 to 12.32)*3 years: 6.64 (0.84 to 12.44)*	Improvements in QOL at 3 years f/u for physical QOL and vitality.
Miller et al. [91](Ohio)	RCT	12 months	N = 206 (81%)	**Intervention**Female 88, Male 16 Age 48.1 (9.7) **Control:**Female 73, Male 29, Age 48.1 (9.1)					Access to a secure electronic messaging system between clinicians and plwMS.	Usual care	Symptom and medical management (symptom)	QOL(Euro- Quality of Life)	Mean difference between intervention and control at end of study:EQ-QOL: 76.3 (± 2.6)**Significant difference, P=0.04.No other between-group differences were found.	Access to messaging system did not lead to the expected improvements in patient outcomes.
Seifi et al. [92](Iran)	Longitudinal cohortstudy		N = 28	Female 18, Male 10, Mean age 38 (24 - 55)^c^					2x 45 min self- care sessions for controlling symptoms.	None	Symptom and medical management (Continence management, diet, exercise, energy preservation)	QOL (WHOQOL)	**Mean difference (standard error) before and after self- care program:**Physical health: -15.02 (2.72)*Psychological: 23.36 (2.42)*Social: -13.54 (2.78)*Living: -8.37 (1.70)**Significant difference (P < 0.001).	Self-care program leads to improved QOL.
Stockl et al. [93](USA)	Observational cohort study	7 months	N = 468	**Intervention**Female 131, Male 25, Age 53.3 (± 10.2) **Specialty pharmacy:**Female 133, Male 23, Age 53.5 (± 10.1) **Retail pharmacy:**Female 127, Male 29, Age 52.9 (± 10.5)	✓ N = 195			11.7 (± 8.8)	DMT program (combined disease self management and medication therapy management) to improve knowledge and maximise therapeutic outcomes.	(1) Retail pharmacyOR(2) speciality pharmacy	Symptom and disease management (Medication)	QOL(SF-12) Disability(MS relapse)	**Mean QOL domains at baseline VS 6 months:**Physical: 37.7 (± 10.1) VS 37.9 (± 10.0)Mental: 48.4 (± 10.4) VS 49.9 (11.1)No significant change, (P>0.05). Relapse: 14% VS 9.3%** Significant change, P=0.03.	DTM program decreased MS relapse rate, but there were no significant changes in QOL.

All data are reported as the mean (±SD) unless otherwise stated. aMean (range), bMedian (IQR), cMedian (range).Expanded disability status scale (EDSS), patient-determined disease steps (PDDS), multiple sclerosis quality of life (LMSQOL), satisfaction with life scale (SWLS), exercise self-efficacy scale (ESES), short form-36 (SF-36), multiple sclerosis impact scale- 29(MSIS-29), quality of life-3 (QOL-3), multiple sclerosis quality of life-54 (MSQOL-54), general health questionnaire-28 (GHQ-28), short form-8 (SF-8), World Health Organization Quality of Life (WHOQOL-B), timed 25 foot walk (T25FW), Hamburg quality of life questionnaire multiple sclerosis (HAQUAMS), annual relapse rate (ARR), multiple sclerosis impact scale (MSIS), short form-12 (SF-12)

### Participant characteristics

The 57 included studies included a total of 5830 individuals diagnosed with MS, with a median of 82 individuals per study (IQR, 110). The majority (95%) of the studies included both female and male participants, with approximately 75% of the participants being female. Five percent of the studies (*n* = 3) were restricted to females [59, 83, 87]. Twenty-one percent (*n* = 12) included only participants with the RRMS phenotype [39, 45, 49, 55, 63, 69–75] and 47% (*n* = 27) included participants with all MS phenotypes. The median age of the participants was 45 years (IQR 9.67), with the majority of studies having mean ages of participants in the 30-or 40-year age group. Of the 14 studies (25%) that included a EDSS score to quantify disability [38, 45, 50, 51, 54, 55, 59, 64, 68, 69, 71, 73–75], the median score was 2.9 (IQR 1.8), suggesting most individuals had a relatively limited disability due to MS. The median disease duration of participants was 8.6 years since MS diagnosis (IQR 4.16).

### Description of outcomes

QOL was most commonly measured at follow-up or post intervention using the MS Quality of Life-54 form (MSQOL-54) in 15 of the 57 studies (26%) [37, 38, 55–57, 62–66, 69, 78–80, 82]. The Short Form-36 was used in a further 12 studies (21%) [42, 53, 59, 74, 76, 81, 83, 84, 86, 88–90]. The most commonly used tool to measure neurologic disability was the EDSS in 14 out of 57 studies (25%) [38, 45, 50, 51, 53, 59, 64, 68, 69, 71, 72, 74–76]. This was followed by the relapse rate (*n* = 7) [46, 70–73, 74, 93], MRI brain scans, including lesion count and volume (*n* = 6) [45, 46, 55, 68, 70, 73] and the PDDS (*n* = 4) [41, 49, 52, 84].

### Qualitative synthesis of individual studies

The most commonly reported intervention dimensions were physical activity and exercise (*n* = 21, 37%) [37–57]; coping, depression, stress, and emotional management (*n* = 10, 18%) [58–67]; diet (*n* = 8, 14%) [68–75], lifestyle and wellbeing (*n* = 8, 14%) [76–83]; fatigue management (*n* = 7, 12%) [84–90] and symptom and medical management (*n* = 3, 5%) [91–93]. Key findings are explored below for each dimension.

#### Physical activity interventions

Of the 21 studies that assessed physical activity, seven reported a significant improvement in QOL domains and only one saw a significant improvement in disability measures among plwMS [37–57]. One study was delivered via multimodal means [43] and the remaining 20 were delivered face-to-face [37–42, 44–57]. The most common QOL domains that saw significant improvement among the seven studies were physical (*n* = 4), mental (*n* = 4), emotional (*n* = 2), energy (*n* = 1), and fatigue (*n* = 1) domains.

Disability was most commonly measured using EDSS (*n* = 6) and lesion characteristics (*n* = 3). The most common intervention activity was general exercise (*n* = 5), followed by aerobic exercise (*n* = 5) and yoga (*n* = 3). Other types include walking, expedition, live-in training programs, resistance training, and inpatient rehabilitation programs.

Among those studies reporting a significant improvement in outcome measures, group-based interventions were a common occurrence, and exercise sessions ranged from to 60–90 minutes in duration.

General exercise interventions ranged from six weeks to 14 months, with typical programs involving team sports, walking, and group activities. Of these, none of the general exercise programs saw a significant change in MS disability, with only one study reporting a significant improvement in QOL for up to 14-months after a combined exercise program.

Of the five studies assessing aerobic exercise [38, 46, 51, 55, 56], four reported significant improvements in QOL subscales [38, 51, 55, 56] including mental, physical, pain, energy, social, sexual function, fatigue, and overall QOL. Two studies reported a significant decrease in the disability measures [46, 55]. One of these studies was restricted to participants with only the RRMS phenotype and included only those prescribed fingolimod as a DMT. Among those studies exploring yoga as an intervention, two reported significant improvements in emotional, energy, and vitality QOL domains at the six month time point.

#### Coping, depression, stress and emotional management interventions

Of the ten studies that explored coping, depression, stress, and emotional self-management strategies [58–67], seven studies were RCT's, all of which reported a significant improvement in QOL post intervention, relative to the controls [59–63, 66, 67]. Controls were not exposed to the interventions and continued with routine standardised MS care. Six were delivered face to face [59, 61–63, 66, 67] and one was delivered via telephone [60]. One of these studies was conducted among adults with a RRMS phenotype only, all others did not restrict the MS phenotype in their inclusion criteria. Physical (*n* = 6) and mental health (*n* = 5)-related QOL were the most common domains reported. A key finding was that significant improvements in QOL domains among the seven interventional studies were reported almost immediately and lasted for up to six months. Few studies have included data on the long-term effects of these interventions (i.e. 12 months and beyond).

Common theories, models, and practical strategies were used in the seven studies that reported a significant improvement in QOL domains. The most popular theory was cognitive behavioural theory (*n* = 3), followed by supportive/positive expressive psychotherapy (*n* = 3), and mindfulness practice (*n* = 2). Two studies explored the effect of the *Social Cognitive Can Do Program* (SCDP) among people with a RRMS phenotype [64, 65]. Each study collectively reported a significant immediate improvement in mental and physical health-related QOL at six months; however, only physical QOL maintained statistical significance at 12-months post intervention.

#### Dietary interventions

The impact of dietary patterns and nutritional supplementation on QOL and disability measures in plwMS was assessed in eight studies. [68–75] Of the three studies that assessed the impact of fish oil supplementation [73– 75], only one reported an immediate improvement in the physical composite score of QOL post intervention relative to a control group [74]. The intervention group received a combination of six fish oil capsules per day (198 mg EPA, 132 mg DHA), 400 units of vitamin E, 1 multivitamin, and 500 mg calcium while following a very low fat diet (i.e. < 15% total energy from fat). The control group was fed a low-cholesterol diet (i.e. < 30% total energy from fat), 400 units of vitamin E, 1 multivitamin, 500 mg calcium, and an olive oil supplement as a placebo. No clinical benefit of fish oil supplementation was found in relation to disability measured using the EDSS or the number of new T1 enhanced and hypo-intensive brain lesions [73, 75].

One study evaluated the effect of probiotic intake and reported a significant improvement in EDSS and general health QOL, favouring the intervention [72].

Only one study assessing the impact of vitamin D supplementation found a significant improvement in mental and health-related subscales [69]. The intervention consisted of patients on interferon-beta as their main DMT, supplementing 50,000 IU vitamin D3 every five days for three months. Controls consumed a placebo. Vitamin D supplementation at 20,000 IU was found to have no significant impact on disability measures via the EDSS or relapse rate [68, 71] however, one study of the same supplemental dose did observe a significant decrease in the number of T1 weighted lesions [68]. Control participants were given a placebo and continued with usual care.

#### Lifestyle and wellbeing

Of the eight studies that assessed lifestyle and/or well-being programs, all were delivered face-to-face, and all reported a significant improvement in QOL, favouring lifestyle self-management courses or exposures [76–83]. The most common QOL domains that saw a significant improvement across the eight studies included mental health (*n* = 7) [76, 78– 83] and physical health (*n* = 6) [76, 78–82] related QOL. All studies included an intervention or exposure to at least two or more self-management dimensions, and the most common combinations included a dietary (*n* = 6), exercise (*n* = 6), and a psychological (emotional, fatigue, stress, coping, support) component (*n* = 6). In two studies, a group-based self-management workshop, delivered by trained healthcare professionals, reported both a short-term (immediate) and long-term (three years) improvement in QOL [80, 82]. A key finding, however, was the sustained improvement in a majority of QOL subscales, including physical health, mental health, pain, and social and overall QOL, for up to five years post intervention [78, 79]. Only one study [83] used a combination of delivery methods including a face-to-face lifestyle program, followed by three months of accountability telephone calls. Improvements in QOL domains, including pain and mental health, were sustained for the duration of the 8-month intervention period.

#### Fatigue management

Of the seven studies that assessed the impact of fatigue management programs on QOL [84–90], four reported a significant improvement in QOL, favouring the fatigue management course. Five were delivered face-to-face [85–87, 89, 90] and two were delivered remotely [84, 88] via teleconference sessions. Of the interventions delivered remotely, both were conducted over a 6-month period however only one reported a significant improvement in physical, vitality and social QOL, favouring the intervention [91–93]. The remaining study comprised of a simpler intervention including only three teleconference sessions and four individually tailored counselling phone calls [88].

Of the interventions delivered face-to-face, three reported a significant improvement in domains of QOL [84–86]. These three studies ranged from six weeks to three years, however they displayed similar participant characteristics, including mean age of participants around 48 years and disease duration of ~ 9.5 years. The most common QOL domains that saw a significant improvement after fatigue and energy conservation courses included vitality, social, mental health, and physical health-related QOL [84–86, 90]. One study that reported no significant improvement after a fatigue intervention accompanied by usual care suggested a longer term follow-up of two years or more may be required to observe a consistent and significant change in QOL [89].

#### Symptom and medical management

The impact of symptom and medical management on QOL was assessed in three studies [92, 94]. Participants followed either a symptom self-care program [91, 92] or program combining medication management and education [93]. Of these three, one study reported a significant improvement in general health-related QOL after a 12 month intervention period, relative to a control group [91]. This study was delivered via an electronic messaging system between plwMS and their clinicians, to enhance symptom control and management. Controls continued their usual MS care. Of the remaining two observational studies, only one revealed a significant improvement in physical, psychological, social, and environmental QOL after a self-care management course [92]. The remaining study found no significant improvement in QOL after a DMT self-management program; however, they reported a 33.6% significant decrease in relapse rate [93].

#### Meta-analysis of studies

Only one self-management dimension met the inclusion criteria for a meta-analysis to calculate pooled estimates of the effect of self-management interventions on homogenous disability outcome measures. An additional two meta-analyses were eligible based purely on a homogenous self-management dimension, QOL domain and summary measure. However, the heterogeneity that was created by comparing different study designs would impact the interrelation of results and a decision to exclude these meta-analyses was made by the authors.

- i. Diet

Five studies, including 286 participants, examined the effect of dietary interventions on disability, using the EDSS [68, 70–72, 75]. Aivo et al. [68] Kouchaki et al. [72] and Zandi- Esfahan et al. [75] reported a reduction in EDSS scores following dietary intervention, while Bitarafan et al. [70] and Kampan et al. [71] did not observe a change in EDSS between the intervention and control arms. None of the results were statistically significant.

**Fig. 2:**
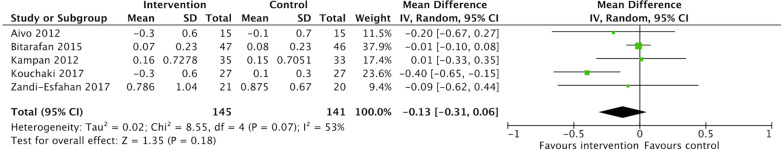
Effect of dietary intervention on mean EDSS difference. Standard deviation (SD), inverse variance (IV), confidence interval (CI)

As demonstrated in Fig. 2, dietary intervention had no statistically significant overall effect on reducing disability as measured by EDSS scores (MD = − 0.13, 95% CI = − 0.31–0.06, Heterogeneity: Tau^2^ = 0.02, Chi^2^ = 8.55, df = 4 (*P* = 0.07); *I*^2^ = 53%, test for overall effect Z = 1.35 (*P* = 0.18). An *I*^*2*^ value of 53% indicated substantial heterogeneity between studies therefore caution is warranted in interpreting these summary estimates. The certainty of the body of evidence by GRADE for dietary interventions was very low, based on downgrading for inconsistency, indirectness, and imprecision. Results of individual GRADE domains are outlined in Additional file 1: data 3.

### Risk of bias in individual studies

The RoB for each included study is presented in Additional file 1: data 2. The RoB ranged from low to high across 35 RCT’s. Overall, nine studies had a RoB [44, 52, 62, 65, 68, 71, 73, 79, 88], 11 had some [46, 50, 51, 60, 67, 69, 75, 76, 83, 84, 90] and 15 had a high RoB [38, 43, 45, 53–56, 59, 61, 63, 66, 74, 86, 89, 91]. The majority (97%) had a low RoB with selection of the reported results [43–46, 48, 50, 51, 53–56, 59, 61–63, 66–76, 83, 86, 88, 89, 91].Thirty-seven percent of studies had some or a high RoB due to the effect of assignment to intervention [38, 43, 46, 50, 53, 55, 59, 61, 63, 66, 89, 90, 91] and 34% of studies had some or a high RoB due to missing outcome data [43, 45, 55, 59, 60, 66, 67, 74, 89, 91].

The RoB in nonrandomised studies of interventions ranged from low to serious. Overall, one study had a low RoB [77], five had a moderate [42, 57, 58, 77, 85] and one had a serious RoB due to intervention classification [49]. All (100%) of studies had a low RoB due to confounding and selective reporting of results. However, 57% (*n* = 4) [49, 57, 58, 82] studies had a moderate RoB due to measurement of the outcome, and 42% (*n* = 3) [58, 82, 85] studies had a moderate RoB in the selection of participants.

Based on the NOS, four of the 13-cohort/case control studies were of good quality [41, 78, 87, 93], three were of satisfactory quality [39, 64, 65], and the remaining six were unsatisfactory [37, 52, 79, 80, 83, 92]. Most studies with unsatisfactory scores did not follow appropriate recruitment methods, assessment of outcomes, and/or adequate follow-up of cohorts. The two cross-sectional studies were of satisfactory quality [47, 48]. None of the studies were appraised as having very good quality, mainly because the selected participants were not representative of the exposed cohort and a poor description of outcome assessments. This was possibly due to the nature of the study design and the inability to blind the participants.

## Discussion

To the best of our knowledge, this is the first review to systematically explore the effectiveness of lifestyle self-management strategies and/or intervention(s) that influence the health and well-being of plwMS, using a common metric of success. For each of the six self-management dimensions (physical activity and exercise; coping, depression, stress, and emotional management; diet, lifestyle, and well-being; fatigue management; and symptom management), varying impacts on participants’ self-reported QOL and objective measures of disability were reported. Qualitative syntheses of results initially appear promising and are reflected in previous work that has explored lifestyle self-management in MS cohorts [11–13]. However, the meta-analysis did not identify any statistically significant effects, which may be a result of the heterogeneity between the included studies and/or due to the small number of studies being meta-analysed [94]. Further to this, the review found that the evidence base for high-quality randomised controlled trials (*n* = 9) designed to improve QOL and reduce objective measures of disability was limited. Therefore, the overall ability to answer the research question: What are the elements of lifestyle self-management strategies and/or interventions that improve the QOL of plwMS patients, was challenged.

The first key finding was that the eight studies that incorporated multicomponent self-management interventions (i.e. lifestyle and well-being programs) [76–83] all reported a significant improvement in self-reported QOL. A reasonable explanation for this finding can be provided through their respective self-management programs that assist participants in gaining skills to control physical symptoms, identify coping strategies to deal with emotional challenges, conserve energy to deal with fatigue, and provide education regarding appropriate dietary choices. As lifestyle is multidimensional, these results were somewhat expected, as the interventions were able to address multiple aspects of care to improve overall QOL. This is consistent with the findings of other studies that have explored multicomponent interventions to improve QOL in chronic disease [95]. Furthermore, these studies used a range of teaching modalities, which have been found to reinforce knowledge comprehension and ensure individual learning styles are addressed [96]. This may have further contributed to the positive improvements in QOL, as self-reported by the participants. Future research should explore the best combinations of self-management dimensions with the greatest effect on improving QOL.

A second key finding was the positive effect of coping, depression, stress, mental health, and/or emotional management strategies in improving the QOL of plwMS [58–67]. This result has been described in the literature for other chronic diseases [97–99]. It is well established that people with chronic illness find it difficult to cope with the physical and/or emotional challenges of their condition [100]. Furthermore, these challenges have been previously reported to significantly affect QOL due to the impact on one’s ability to work, participate in leisure activities, and engage in social functions. Therefore, it was anticipated that a program equipped with plwMS with the confidence to self-manage the emotions of their diagnosis and develop coping strategies to deal with stress would reduce the burden of their condition and improve QOL. This finding is consistent with a previous systematic review exploring the effectiveness of self-management to improve depression, anxiety and QOL among plwMS [11]. As reported by Kidd et al. effective interventions included principles of the cognitive behavioural theory, in 30% of the studies. One meta-analysis reported that incorporating cognitive behavioural theory in interventions for plwMS is effective in improving QOL domains [11, 101]. The present review has validated this finding.

Fatigue is one of the most common symptoms and burdens approximately 75% of plwMS [102]. It is well established that fatigue is independently associated with impaired QOL in MS, suggesting that recognising and treating fatigue can potentially improve overall life quality [102, 103]. Therefore, interventions encapsulating self-management of fatigue would significantly improve QOL. However, this assumption was only reflected in four out of the seven studies exploring fatigue self-management with interventions ranging from six weeks to three years. One possible reason for these inconsistencies is that behavioural change takes time and, therefore, longer term follow up of two years or more may be required to observe a consistent and significance change in QOL. This was observed in a similar review of fatigue self-management in patients with chronic health conditions such as chronic fatigue syndrome, rheumatoid arthritis, and cancer, suggesting longer follow up times are needed [104].

The pooled estimate of the effect of dietary interventions among the five studies indicated no significant reduction in disability measures reported through the EDSS. According to Higgins et al. [105] the *I*^2^ value indicated substantial heterogeneity; therefore, the results should be interpreted with caution. This may be attributed to the substantial variation in the study design, intervention, and methodology among the six included studies. Due to the scarcity of evidence confirming particular food(s) or dietary patterns that are effective in reducing the symptom burden of MS, it was anticipated that no significant effect would be found in this review. These results are consistent with a 2020 Cochrane review, which indicated that there is an insufficient level of evidence to determine whether dietary supplementation and certain dietary patterns have an impact on MS-related outcomes [106]. However, a promising effect of diet on improving QOL domains was observed in these studies. Despite this, high-quality, food-focused clinical trials exploring dietary interventions as a self-management strategy are still warranted.

The reported effect of physical activity on outcome measures was largely inconsistent across 21 studies. In total, 50% (*n* = 9/18) of studies reported a significant improvement in QOL, and only 38% (*n* = 3/8) of studies reported a significant improvement in disability measures, such as decreased EDSS score, lesion count, and annualised relapse rate. Previous studies have found that plwMS who participate in regular physical activity report higher QOL [107]. Therefore, if MS participants in these studies were already actively partaking in planned physical activity and exercise, it was anticipated that a ceiling effect was observed [108]. That is, participants were involved in regular physical activity as a self-management strategy prior to the intervention; therefore, there was little opportunity for improvements in QOL to be observed post intervention [108]. Again, future reviews should aim to quantitatively synthesise homogenous results via a meta-analysis to comment on overall effectiveness.

Despite these findings, it is important to note the limitations of this review. Multiple scientific databases were searched using specific terms and truncations. However, the review may have excluded potential relevant studies due to the subjectivity of what constitutes self-management.” Additionally, excluding non-English studies, case studies, review articles, feasibility and pilot studies, protocols and grey literature, conference abstracts, editorials, and monographs may mean that the analysis in the review is limited.

Despite rigorous inclusion criteria, the heterogeneity of the included studies was still evident, therefore, a qualitative synthesis of the included studies was conducted. Several studies recruited only female participants or only individuals with a relapsing–remitting MS phenotype. Multiple QOL questionnaires that differed in their reported domains of QOL were included, making cross-study comparisons difficult. Moreover, a number of studies did not report sufficient outcome and summary measures detailed to their intervention; therefore, quantitative analysis through a meta-analysis was not possible for a majority of the included studies and their respective self-management dimensions. This prevents a definitive answer to the PICO question, without further research.

## Conclusion

Multicomponent self-management interventions that incorporate coping, stress, depression, emotional management, multimodal delivery methods, and cognitive behavioural theory principals were common elements of self-management interventions that improved the QOL of plwMS. Dietary intervention had no statistically significant overall effect on reducing MS disability, (*P* = 0.18). The overall effect of physical activity on these measures was inconsistent.

This review revealed a significant gap in the literature, warranting high-quality, large-scale experimental, and observational studies that address the research question. Therefore, these results should be interpreted with caution, and care should be taken in clinical applications. Heterogeneity continues to limit the ability to pool the effects from a large number of studies. A future review addressing this as evidence-based growth is warranted. The question of the best combination of lifestyle self-management dimensions to improve QOL remains. Therefore, future studies should not only address one dimension, but also explore different combinations of plwMS.

## Supplementary Information

**Additional file 1**: **Data 1**. Database search strategy and search results. **Data 2**. A. Cochrane risk of bias assessment for included randomised controlled trials (*n* = 35). B. Newcastle Ottawa risk of bias scale (NOS) for case control and cohort studies (*n* = 13). C. Newcastle Ottawa risk of bias scale (NOS) adapted for cross- sectional studies (*n* = 2) D. Risk of bias in non- randomised studies of interventions (ROBINS-I) (*n* = 7). **Data 3**. GRADE for assessing the certainty of the body of evidence.

## Data Availability

The datasets analysed during the current review are available from the corresponding author on reasonable request.

## Abbreviations

MS: Multiple sclerosis
DMT: Disease-modifying therapy
plwMS: People living with MS
QOL: Quality of life
EDSS: Expanded disability status scale
PDDS: Patient-determined disease steps
RoB: Risk of bias
RCT: Randomised control trial
NOS: Newcastle–Ottawa scale
NHMRC: National health and medical research council
