# Supplementary material for: Understanding lifestyle self-management regimens that improve the life quality of people living with multiple sclerosis: a systematic review and meta-analysis
Source: Health Qual Life Outcomes. 2022 Nov 25;20:153. doi: 10.1186/s12955-022-02046-1 (PMC9700996; doi:10.1186/s12955-022-02046-1)
Supplement: Supplementary file 1 — Additional file 1: Data 1. Database search strategy and search results. Data 2. A. Cochrane risk of bias assessment for included randomised controlled trials (n = 35). B. Newcastle Ottawa risk of bias scale (NOS) for case control and cohort studies (n = 13). C. Newcastle Ottawa risk of bias scale (NOS) adapted for cross- sectional studies (n = 2) D. Risk of bias in non- randomised studies of interventions (ROBINS-I) (n = 7). Data 3. GRADE for assessing the certainty of the body of evidence. [file 12955_2022_2046_MOESM1_ESM.docx]

# **Supplemental data 1:** *Database search strategy and search results*

**Cochrane Library (Search field: All text, word variations were searched) n=88**

## "Multiple sclerosis" AND "Self-manag*" OR “lifestyle self-manag*” OR "self care" OR "patient participat*" OR lifestyle OR "patient care plan*" OR "disease manag*" OR "self-efficacy" OR "self monitor*" OR "health behavio*" OR “health education” OR “self management skills” AND Nutrition OR “nutrition* therapy” OR “diet therapy” OR diet OR exercise OR "physical activity" OR activity OR psycholo* OR meditat* OR relaxat* OR smok* OR rehab* OR “stress manag*” OR “fatigue manag*” OR “supplement*” OR sleep*

## AND "Disease progress*" OR "Expanded Disability Status Scale" OR EDSS OR "Multiple sclerosis functional composite" OR MSFC OR “lesion burden” OR “lesion volume” OR “number of lesion*” OR “brain atrophy” OR “number of relapses” OR “relapse rate” OR "no evidence of progression" OR NEP OR "nine-hole peg test" OR "timed 25-foot walk" OR PDDS OR “Patient Determined Disease Steps” OR “quality of life” OR “MS quality of life inventory” OR MSQLI OR “modified fatigue impact scale” OR MFIS OR “Multiple Sclerosis Impact Scale” OR “Guys neurological disability scale” OR GNDS OR MRI OR “magnetic resonance imaging” OR “fatigue severity scale” OR FSS

**MEDLINE n= 590**

**CINAHL n=295**

## **#1** (MM “Multiple sclerosis+”) OR "Multiple sclerosis"

## **#2** (MM “Self-Management+”) OR "Self-manag*" OR “lifestyle self-manag*” OR "self care" OR "patient participat*" OR lifestyle OR "patient care plan*" OR "disease manag*" OR "self-efficacy" OR "self monitor*" OR "health behavio*" OR “health education” OR “self management skills”

## **#3** (MM “Diet therapy+”) OR (MM “Nutrition therapy+”) Nutrition OR “nutrition* therapy” OR “diet therapy” OR diet OR exercise OR "physical activity" OR activity OR psycholo* OR meditat* OR relaxat* OR smok* OR rehab* OR “stress manag*” OR “fatigue manag*” OR “supplement*” OR sleep*

## **#4** (MM “Disease Progression+”) OR "Disease progress*" OR "Expanded Disability Status Scale" OR EDSS OR "Multiple sclerosis functional composite" OR MSFC OR “lesion burden” OR “lesion volume” OR “number of lesion*” OR “brain atrophy” OR “number of relapses” OR “relapse rate” OR "no evidence of progression" OR NEP OR "nine-hole peg test" OR "timed 25-foot walk" OR PDDS OR “Patient Determined Disease Steps” OR “quality of life” OR “MS quality of life inventory” OR MSQLI OR “modified fatigue impact scale” OR MFIS OR “Multiple Sclerosis Impact Scale” OR “Guys neurological disability scale” OR GNDS OR MRI OR “magnetic resonance imaging” OR “fatigue severity scale” OR FSS

**#1 AND #2 AND #3 AND #4**

**PubMed (Searched all fields) n= 691**

"Multiple sclerosis" AND "Self-manag*" OR “lifestyle self-manag*” OR "self care" OR "patient participat*" OR lifestyle OR "patient care plan*" OR "disease manag*" OR "self-efficacy" OR "self monitor*" OR "health behavio*" OR “health education” OR “self management skills” AND Nutrition OR “nutrition* therapy” OR “diet therapy” OR diet OR exercise OR "physical activity" OR activity OR psycholo* OR meditat* OR relaxat* OR smok* OR rehab* OR “stress manag*” OR “fatigue manag*” OR “supplement*” OR sleep* AND "Disease progress*" OR "Expanded Disability Status Scale" OR EDSS OR "Multiple sclerosis functional composite" OR MSFC OR “lesion burden” OR “lesion volume” OR “number of lesion*” OR “brain atrophy” OR “number of relapses” OR “relapse rate” OR "no evidence of progression" OR NEP OR "nine-hole peg test" OR "timed 25-foot walk" OR PDDS OR “Patient Determined Disease Steps” OR “quality of life” OR “MS quality of life inventory” OR MSQLI OR “modified fatigue impact scale” OR MFIS OR “Multiple Sclerosis Impact Scale” OR “Guys neurological disability scale” OR GNDS OR MRI OR “magnetic resonance imaging” OR “fatigue severity scale” OR FSS

**Scopus (Searched all fields) n=6**

## "Multiple sclerosis" AND "Self-manag*" OR “lifestyle self-manag*” OR "self care" OR "patient participat*" OR lifestyle OR "patient care plan*" OR "disease manag*" OR "self-efficacy" OR "self monitor*" OR "health behavio*" OR “health education” OR “self management skills” AND Nutrition OR “nutrition* therapy” OR “diet therapy” OR diet OR exercise OR "physical activity" OR activity OR psycholo* OR meditat* OR relaxat* OR smok* OR rehab* OR “stress manag*” OR “fatigue manag*” OR “supplement*” OR sleep*

## AND "Disease progress*" OR "Expanded Disability Status Scale" OR EDSS OR "Multiple sclerosis functional composite" OR MSFC OR “lesion burden” OR “lesion volume” OR “number of lesion*” OR “brain atrophy” OR “number of relapses” OR “relapse rate” OR "no evidence of progression" OR NEP OR "nine-hole peg test" OR "timed 25-foot walk" OR PDDS OR “Patient Determined Disease Steps” OR “quality of life” OR “MS quality of life inventory” OR MSQLI OR “modified fatigue impact scale” OR MFIS OR “Multiple Sclerosis Impact Scale” OR “Guys neurological disability scale” OR GNDS OR MRI OR “magnetic resonance imaging” OR “fatigue severity scale” OR FSS

**Supplemental data 2.**

**A.** *Cochrane risk of bias assessment for included randomised controlled trials (n=35)*

| **Reference** | **Randomisation process** | **Deviations from the intended interventions**  **(Effect of assignment to intervention)** | **Missing outcome data** | **Measurement of the outcome** | **Selection of the reported result** | **Overall** |
| --- | --- | --- | --- | --- | --- | --- |
| Stuifbergen et al, 2003 (USA) | Low | Low | Low | Some | Low | **Some** |
| Pilutti et al, 2014. (Urbana) | Low | Low | Low | Low | Low | **Low** |
| Graziano et al, 2014. (Italy) | Low | Low | Low | Low | Low | **Low** |
| Sangelaji et al 2014 (Iran) | Some | Low | Low | Some | Low | **High** |
| Ennis et al, 2006 (UK) | Low | Low | Low | Some | Low | **Some** |
| Mathiowetz et al. 2005 (Minneapolis) | Low | Some | Low | Some | Low | **High** |
| Savsek et al, 2021 (Slovenia) | Some | Some | High | Low | Low | **High** |
| Kjolhede et al, 2018 (Denmark) | Low | Low | High | Some | Low | **High** |
| Jongen et al. 2019 (Netherlands) | Low | Some | Some | Low | Low | **High** |
| Khan et al, 2008 (Australia) | Low | Low | Low | Low | Low | **Low** |
| Finlayson et al, 2011 (USA) | Low | Low | Low | Low | Some | **Some** |
| Carter et al, 2014 (UK) | Low | High | Low | Low | Low | **High** |
| Plow et al, 2019 (Ohio) | Low | Low | Low | Low | Low | **Low** |
| Langeskov- Christensen et al, 2021. (Denmark) | Low | Some | Low | Low | Low | **Some** |
| Ehde et al, 2015 (US) | Low | Low | Some | Low | Low | **Some** |
| Thomas et al, 2014 (UK) | Low | Some | Low | Some | Low | **Some** |
| Lincoln et al, 2011 (UK) | Low | Low | Some | Low | Low | **Some** |
| Flachenecker et al, 2020 (Germany) | Low | High | High | Low | Low | **High** |
| Miller et al, 2011 (Ohio) | Low | Some | Low | Some | Low | **High** |
| Mutluay et al, 2007 (Turkey) | Low | Some | Low | Low | Low | **Some** |
| Zandi- Esfahan et al, 2017. (Iran) | Low | Low | Low | Low | Low | **Low** |
| Weinstock-Guttman et al, 2005 (NY) | Some | Low | High | Low | Low | **High** |
| Thomas et al, 2013 (UK) | Low | Some | Some | Low | Low | **High** |
| Avio et al, 2012 (Finland) | Low | Low | Low | Low | Low | **Low** |
| Petjan et al, 1996 (UT) | Some | Low | Low | Low | Low | **Some** |
| Sutherland et al, 2001 (Australia) | Some | Low | Low | Some | Low | **High** |
| Kampan et al, 2012 (Norway) | Low | Low | Low | Low | Low | **Low** |
| Hart et al, 2005 (USA) | Some | Some | Low | Some | Low | **High** |
| Kouchaki et al, 2017 (Iran) | Low | Low | Low | Low | Low | **Low** |
| Oken et al, 2004 (Portland) | Some | Some | Low | Low | Low | **High** |
| Ashtari et al, 2016 (Iran) | Some | Low | Low | Low | Low | **Some** |
| Bitarafan et al, 2015. (Iran) | Some | Low | Low | Low | Low | **Some** |
| Torkildsen et al, 2012 (Norway) | Low | Low | Low | Low | Low | **Low** |
| Besharat et al, 2017 (USA) | Some | Some | High | Some | Low | **High** |
| Grossman et al, 2010 (Switzerland) | Low | Some | Low | Some | Low | **High** |

**B.** *Newcastle Ottawa risk of bias scale (NOS) for case control and cohort studies (n= 13).*

|  | **SELECTION** | | | | **COMPARABILITY** | **OUTCOME** | | | **TOTAL SCORE** |
| --- | --- | --- | --- | --- | --- | --- | --- | --- | --- |
|  | **Representativeness of exposed cohort**  **(Max: ★)** | **Selection of the non-exposed cohort**  **(Max: ★)** | **Ascertainment of exposure**  **(Max: ★)** | **Outcome of interest not present at start of study**  **(Max: ★)** | **Comparability of cohorts on the basis of design or analysis**  **(Max: ★★)** | **Assessment of outcome**  **(Max: ★)** | **Was follow up long enough for outcomes to occur**  **(Max: ★)** | **Adequacy of follow up cohorts**  **(Max: ★)** |  |
| D'hooghe et al, 2014  (Belgium) |  |  | **★** | **★** | **★** |  |  | **★** | **★★★★ (4)** |
| Hadhkiss Et al. 2013 (Australia) | **★** |  | **★** | **★** |  |  | **★** |  | **★★★★★ (5)** |
| Li et al, 2010  (Australia) |  |  |  | **★** | **★** |  | **★** |  | **★★★ (3)** |
| Seifi et al,  2018 (Tehran) |  |  |  | **★** | **★★** |  |  |  | **★★★ (3)** |
| Jongen et al. 2014  (Netherlands) | **★** |  | **★** | **★** |  |  |  | **★** | **★★★★ (4)** |
| Marck et al, 2018  (Australia) |  |  | **★** | **★** |  |  | **★** |  | **★★★ (3)** |
| Beatus et al, 2002  (USA) |  |  |  | **★** | **★** |  |  |  | **★★ (2)** |
| Stockl et al, 2010  (USA) |  | **★** |  | **★** | **★★** |  | **★** | **★** | **★★★★★★ (6)** |
| Mulligan et al, 2016 (NZ) | **★** | **★** | **★** | **★** |  |  | **★** | **★** | **★★★★★★ (6)** |
| Frasczewskiet al, 2020 (USA) | **★** |  |  | **★** | **★** | **★** | **★** | **★** | **★★★★★★ (6)** |
| Jongen et al. 2016 (Netherlands) | **★** |  | **★** | **★** | **★** |  |  |  | **★★★★ (4)** |
| Frasczewskiet al, 2018 (USA) |  |  |  |  |  | **★** |  |  | **★ (1)** |
| Ng et al, 2013 (Canada) | **★** |  |  | **★** |  |  | **★** |  | **★★★ (3)** |

**C.** *Newcastle Ottawa risk of bias scale (NOS) adapted for cross- sectional studies (n=2)*

|  | **SELECTION** | | | | **COMPARABILITY** | **OUTCOME** | | **TOTAL SCORE** | |
| --- | --- | --- | --- | --- | --- | --- | --- | --- | --- |
|  | **Representativeness of the sample**  **(Max: ★)** | **Sample size**  **(Max: ★)** | **Non- respondents**  **(Max: ★)** | **Ascertainment of the exposure**  **(Max: ★★)** | **Comparability subjects in different outcome groups on the basis of design or analysis. Confounding factors controlled**  **(Max: ★★)** | **Assessment of outcome.**  **(Max: ★★)** | **Statistical test**  **(Max: ★)** |  |  |
| Motl et al.2009 (USA) | **★** |  |  | **★** | **★** |  | **★** | **★★★★ (4)** |  |
| Motl et al 2007 (USA) | **★** |  |  | **★** | **★** |  | **★** | **★★★★ (4)** |  |

**D.** *Risk of bias in non- randomised studies of interventions (ROBINS-I) (n=7)*

| **Reference** | **Confounding** | **Selection** | **Intervention classification** | **Deviation from intervention** | **Missing data** | **Measurement of outcome** | **Selective reporting of results** | **Overall** |
| --- | --- | --- | --- | --- | --- | --- | --- | --- |
| Feys et al. 2013 (Belgium) | Low | Low | Low | Low | Moderate | Low | Low | **Moderate** |
| Sahebalzamani et al. 2012 (Iran) | Low | Moderate | Low | Moderate | Low | Moderate | Low | **Moderate** |
| Feicke et al.2014 (Germany) | Low | Low | Low | Low | Low | Low | Low | **Low** |
| Motl et al, 2011 (USA) | Low | Low | Serious | Low | Low | Moderate | Low | **Serious** |
| Vasudevan et al, 2021 (India) | Low | Low | Low | Moderate | Moderate | Moderate | Low | **Moderate** |
| Mathiowetz et al. 2001 (USA) | Low | Moderate | Low | Low | Low | Low | Low | **Moderate** |
| Abolghasemi et al, 2016 (Iran) | Low | Moderate | Low | Low | Low | Moderate | Low | **Moderate** |

**Supplemental data 3.** *GRADE for assessing the certainty of the body of evidence.*

| **Component** | **Comment** | **Level** |
| --- | --- | --- |
| **Risk of bias** | Only one meta-analysis of 5 RCT’s were included in the review. Majority (n=4/5) of the studies had a low RoB and one had some RoB due to the randomisation procedures.   - Most information is studies at a low RoB. - Potential limitations are unlikely due to lower confidence in the estimate of effect, **therefore, not downgraded.** | **High**  **= High** |
| **Inconsistency** | The *I^2^* statistic was used to judge inconsistency in the one meta-analysis. The meta-analysis showed a non-significant test for heterogeneity (P=0.18) and substantial *I^2^* of 53%.  Heterogeneity was categorised according to the Cochrane guidelines: (1) *I*^2^= 0% to 40%: low heterogeneity; (2) *I*^2^= 30% to 60%: moderate heterogeneity; (3) *I*^2^= 50% to 90%, substantial heterogeneity; and (4) *I*^2^= 75% to 100%: considerable heterogeneity.^16^  Heterogeneity may be explained by subjective interpretation of ‘self- management,’ participant variability (i.e. MS phenotype, disease duration, disease modifying therapies) and intervention type (what type of supplement was used).  **Therefore, the evidence quality has been downgraded one level.** | **High**  **= Moderate** |
| **Indirectness** | PICO question: *What is the effect of lifestyle self-management strategies and/or interventions on QOL and/or disability in plwMS?*  Population= Evidence regarding participants is direct to the PICO question.  Intervention= Evidence regarding self-management interventions (dietary intervention) is direct to the PICO question.  Comparator= Evidence regarding comparator or control groups is direct to the PICO question.  Outcome= Expanded disability status scale (EDSS) scores are an indirect measure of MS disability and, therefore, may be used as a surrogate endpoint. The use of surrogate markers, therefore, **downgraded the quality of evidence by one level.** | **Moderate**  **= Low** |
| **Imprecision** | This meta-analysis was conducted with relatively few participants (n= 286) and, therefore, has a wide confidence interval (CI= -0.31 to 0.06) meaning that the uncertainty of the true effect size is greater.^a^ Therefore, evidence quality has been **downgraded by one level.** | **Low**  **=Very low** |
| **Publication bias** | No evidence of industry sponsored studies. In addition, all studies had a low RoB under the Cochrane RoB selective outcome reporting domain. **Therefore, the evidence quality was not downgraded.** | **Very low**  **= Very low** |
| **OVERALL GRADE** | **Very low**  **We have very little confidence in the effect estimate: The true effect is likely to be substantially different from the estimate of effect.** | |

^a^ Schunemann HJ, Vist GE, Higgins JPT, Santesso N, Deeks JJ, Glasziou P, et al. Chapter 15: Interpreting results and drawing conclusions. In: Higgins JPT, Thomas J, Chandler J, Cumpston M, Li T, Page MJ, Welch VA (editors). *Cochrane Handbook for Systematic Reviews of Interventions*version 6.3 (updated February 2022). Cochrane, 2022. Available from [www.training.cochrane.org/handbook](http://www.training.cochrane.org/handbook).
